# Genetic Risk Underlying Psychiatric and Cognitive Symptoms in Huntington’s Disease

**DOI:** 10.1016/j.biopsych.2019.12.010

**Published:** 2020-05-01

**Authors:** Natalie Ellis, Amelia Tee, Branduff McAllister, Thomas Massey, Duncan McLauchlan, Timothy Stone, Kevin Correia, Jacob Loupe, Kyung-Hee Kim, Douglas Barker, Eun Pyo Hong, Michael J. Chao, Jeffrey D. Long, Diane Lucente, Jean Paul G. Vonsattel, Ricardo Mouro Pinto, Kawther Abu Elneel, Eliana Marisa Ramos, Jayalakshmi Srinidhi Mysore, Tammy Gillis, Vanessa C. Wheeler, Christopher Medway, Lynsey Hall, Seung Kwak, Cristina Sampaio, Marc Ciosi, Alastair Maxwell, Afroditi Chatzi, Darren G. Monckton, Michael Orth, G. Bernhard Landwehrmeyer, Jane S. Paulsen, Ira Shoulson, Richard H. Myers, Erik van Duijn, Hugh Rickards, Marcy E. MacDonald, Jong-min Lee, James F. Gusella, Lesley Jones, Peter Holmans

**Affiliations:** a Cardiff University School of Medicine, UHW Main Building, Heath Park, Cardiff, United Kingdom; b Medical Research Council Centre for Neuropsychiatric Genetics and Genomics, Division of Psychological Medicine and Clinical Neurology, School of Medicine, Cardiff University, Cardiff, United Kingdom; c Cardiff University Brain Research Imaging Centre, Cardiff University, Cardiff, United Kingdom; d All Wales Medical Genetics Service, Institute of Medical Genetics, University Hospital Wales, Cardiff, United Kingdom; e Department of Targeted Intervention, Division of Surgery and Interventional Science, Faculty of Medical Science, University College of London, London, United Kingdom; f Molecular Neurogenetics Unit, Center for Genomic Medicine, Massachusetts General Hospital, Boston, Massachusetts; g Department of Neurology, Harvard Medical School, Boston, Massachusetts; h Department of Genetics, Blavatnik Institute, Harvard Medical School, Boston, Massachusetts; i Department of Neurology and Genome Science Institute, Boston University School of Medicine, Boston, Massachusetts; j Medical and Population Genetics Program, the Broad Institute of M.I.T. and Harvard, Cambridge, Massachusetts; k HudsonAlpha Institute for Biotechnology, Huntsville, Alabama; l Department of Biostatistics, College of Public Health, University of Iowa, Iowa City, Iowa; m Department of Psychiatry, Roy and Lucille Carver College of Medicine, University of Iowa, Iowa City, Iowa; n Department of Neurology, Roy and Lucille Carver College of Medicine, University of Iowa, Iowa City, Iowa; o Department of Pathology and Cell Biology and the Taub Institute for Research on Alzheimer’s Disease and the Aging Brain, Columbia University Medical Center, New York, New York; p Department of Neurology, University of Rochester Medical Center, Rochester, New York; q CHDI Foundation, Princeton, New Jersey; r Institute of Molecular, Cell and Systems Biology, College of Medical, Veterinary and Life Sciences, University of Glasgow, Glasgow; v National Centre for Mental Health, Birmingham and Solihull Mental Health NHS Foundation Trust, Birmingham, United Kingdom; w College of Medical and Dental Sciences, University of Birmingham, Birmingham, United Kingdom; s Department of Neurology, University of Ulm, Ulm, Germany; t Department of Psychiatry, Leiden University Medical Centre, Leiden, Netherlands; u Mental Health Care Centre Delfland, Delft, Netherlands

**Keywords:** Cognition, Depression, Huntington’s disease, Polygenic risk, Psychiatric, Schizophrenia

## Abstract

**Background:**

Huntington’s disease (HD) is an inherited neurodegenerative disorder caused by an expanded CAG repeat in the *HTT* gene. It is diagnosed following a standardized examination of motor control and often presents with cognitive decline and psychiatric symptoms. Recent studies have detected genetic loci modifying the age at onset of motor symptoms in HD, but genetic factors influencing cognitive and psychiatric presentations are unknown.

**Methods:**

We tested the hypothesis that psychiatric and cognitive symptoms in HD are influenced by the same common genetic variation as in the general population by 1) constructing polygenic risk scores from large genome-wide association studies of psychiatric and neurodegenerative disorders and of intelligence and 2) testing for correlation with the presence of psychiatric and cognitive symptoms in a large sample (*n* = 5160) of patients with HD.

**Results:**

Polygenic risk score for major depression was associated specifically with increased risk of depression in HD, as was schizophrenia risk score with psychosis and irritability. Cognitive impairment and apathy were associated with reduced polygenic risk score for intelligence.

**Conclusions:**

Polygenic risk scores for psychiatric disorders, particularly depression and schizophrenia, are associated with increased risk of the corresponding psychiatric symptoms in HD, suggesting a common genetic liability. However, the genetic liability to cognitive impairment and apathy appears to be distinct from other psychiatric symptoms in HD. No associations were observed between HD symptoms and risk scores for other neurodegenerative disorders. These data provide a rationale for treatments effective in depression and schizophrenia to be used to treat depression and psychotic symptoms in HD.

SEE COMMENTARY ON PAGE e25

Huntington’s disease (HD) is an inherited neurodegenerative disorder caused by an expanded CAG repeat in *HTT*. A clinical diagnosis is typically made via a movement disorder, but nearly all participants experience progressive cognitive decline, and many exhibit behavioral and psychiatric symptoms (1). Depression, irritability, obsessive and compulsive symptoms, apathy, and psychosis all occur at rates higher than seen in the non-HD population, though they are not universal in HD (2). Psychiatric symptoms are often present before motor symptoms become manifest. Age at motor onset of HD is determined both by the length of the CAG repeat tract in *HTT* and by other genetic variants in the genome (3, 4, 5). Despite the fact that age at motor onset measures only one specific facet of the pathological process (1), it has been widely used to identify genetic modifiers in HD, while genetic influences on behavioral and neuropsychiatric symptoms in HD have not been systematically investigated. Small studies have shown familial aggregation of psychosis in HD (6,7) with weak evidence for the influence of specific candidate genes (8).

Common genetic variation contributes to the risk of developing schizophrenia (9), bipolar disorder (BPD) (10), major depressive disorder (MDD) (11), and attention-deficit/hyperactivity disorder (ADHD) (12). There is significant shared genetic risk among these psychiatric disorders (13).

Increased general intelligence—a measure of cognitive function—has been shown to be genetically correlated with reduced risk of Alzheimer’s disease (AD), ADHD, and schizophrenia (14). As in HD, there are substantially increased levels of psychiatric symptoms in many neurological diseases. For instance 50% of those with AD (15) and up to 75% of those with Parkinson’s disease (PD) develop psychotic symptoms (16,17). In dementia with Lewy bodies, visual hallucinations are a core clinical feature seen in 80% of patients (18). AD with psychosis is heritable (19,20), although increasing polygenic risk score (PRS) for schizophrenia was associated with reduced risk of psychosis in AD (21).

Given the increased frequency of neuropsychiatric and cognitive symptoms in HD, it is of interest to test for genetic overlap of these symptoms with psychiatric and neurodegenerative disorders and intelligence. This was done by constructing PRSs using the latest available genome-wide association studies for these disorders and testing these for correlation with the presence of neuropsychiatric and cognitive symptoms in HD.

## Methods and Materials

### HD Participants and Phenotypes

The HD participants in this analysis were part of the European REGISTRY study (22) or its successor, the international Enroll-HD (23) observational study of HD. REGISTRY was a multisite, prospective, observational study, which collected phenotypic data (2003–2013) for more than 13,000 participants, mostly HD gene carriers with manifest disease. Enroll-HD is an expanded and modified version of the REGISTRY study and is international: to date it has over 16,000 participants (some of whom rolled over from REGISTRY) from 19 nations. All experiments were performed in accordance with the Declaration of Helsinki, and ethical approval for the REGISTRY and Enroll-HD studies including written informed consent of all participants was obtained. This study was approved by Cardiff University School of Medicine Research Ethics Committee.

There were 6278 individuals with manifest HD defined by motor onset from REGISTRY (*n* = 4986) and Enroll-HD (*n* = 1292) with appropriate quality-controlled genome-wide association study (GWAS) data as described elsewhere (5). We examined 7 symptoms—depression, irritability, psychosis, apathy, violent/aggressive behavior, perseverative/obsessive behavior, and cognitive impairment—from the clinical characteristics questionnaire in REGISTRY and Enroll-HD. The clinical characteristics questionnaire asks if a sign or symptom has ever been experienced by a subject (see Supplement). At least one clinical characteristics questionnaire symptom endorsement (positive or negative) was recorded in 5854 participants (4563 REGISTRY, 1291 Enroll-HD). We removed 133 individuals with a comorbid diagnosis of BPD, schizophrenia, schizotypy, or schizoaffective disorder (since these are likely to share risk genes for psychiatric disorders independently of their HD status). We also removed 1 member (561 individuals) of each pair of first- or second-degree relatives (identity by descent probability > 0.25) to minimize the correlation between individuals due to cryptic relatedness. This left 5160 participants in the final analysis (Supplemental Figure S1).

### Genetic Analysis

For each individual in the HD dataset, genetic risk for each psychiatric/neurodegenerative disorder was captured by a PRS (24). A PRS is defined as the sum of the number of minor alleles across a set of single nucleotide polymorphisms (SNPs), each weighted by the corresponding risk of that allele for the psychiatric/neurodegenerative disorder observed in a “training” GWAS. The set of SNPs used to calculate the PRS was chosen to be present in both the training GWAS and the HD dataset, to be in approximate linkage equilibrium, and to capture as much of the association signal in the training GWAS as possible. Following the procedure outlined by the Psychiatric Genomics Consortium (PGC) (9), this was achieved by using PLINKv1.9 (25) to select the most significant SNP in the training GWAS, removing all SNPs within 500 kb that were in linkage disequilibrium (*r*^2^ > .1) with it, then moving to the next most significant remaining SNP and repeating the process. Analysis was restricted to SNPs with minor allele frequency > 0.01 that were well imputed (*r*^2^ between allele dosages and the unknown true genotypes > .9) in the HD dataset and (where this information was available) in the training GWAS. SNPs were selected for inclusion into the PRS by applying criteria to their *p* values in the training set. Since the optimal criterion was not known a priori, we applied 6 different *p*-value cutoffs (*p* < .0001, *p* < .001, *p* < .01, *p* < .05, *p* < .5, *p* < 1). Effects of population stratification were removed by regressing the PRS on 20 principal components. The residuals from this regression were standardized to enable comparison of effect sizes across cutoffs.

Nine large publicly available sets of GWAS summary statistics were used for training (Supplemental Table S1). These comprised 5 GWASs of psychiatric disorders from the PGC [schizophrenia, BPD, ADHD, autism spectrum disorder, and obsessive-compulsive disorder (OCD) (9,10,12,26,27)], a meta-analysis of the PGC and UK Biobank MDD samples (11), 2 neurodegenerative disorders [late-onset AD (28) and PD (29)], and a large GWAS of general intelligence (14). The major histocompatibility complex region was removed from the schizophrenia GWAS owing to a strong schizophrenia signal and long-range linkage disequilibrium potentially biasing the PRS (9). Summary statistics for the PGC GWAS (including the PGC and UK Biobank MDD meta-analysis) are available from https://www.med.unc.edu/pgc/results-and-downloads; those for the AD and intelligence GWASs are available from https://www.ebi.ac.uk/gwas/downloads/summary-statistics; and those from the PD GWAS (omitting 23andMe samples) are available from https://drive.google.com/drive/folders/10bGj6HfAXgl-JslpI9ZJIL_JIgZyktxn.

Associations were tested between all 9 PRSs and all 7 symptoms at each PRS cutoff. The following *p*-value criteria were used to define significance correcting for multiple testing: 7.94 × 10^−4^ (Bonferroni corrected for 7 symptoms tested on 9 disease PRSs, a total of 63 tests), 1.32 × 10^−4^ (Bonferroni corrected for 63 tests × 6 PRS cutoffs).

We also defined 27 PRS-symptom comparisons as primary hypotheses of interest, reflecting prior associations in the general population between the phenotypes and the diseases from which the PRS were derived. These were 1) MDD with depression, irritability, and apathy; 2) PD with depression and irritability; 3) schizophrenia with depression, irritability, psychosis, apathy, and violent/aggressive behavior; 4) ADHD with irritability, violent/aggressive behavior, and cognitive impairment; 5) autism spectrum disorder with irritability, psychosis, and perseverative/obsessive behavior; 6) OCD with perseverative/obsessive behavior; 7) AD with depression, irritability, apathy, violent/aggressive behavior, and cognitive impairment; 8) PD with depression, irritability, apathy, and cognitive impairment; and 9) intelligence with cognitive impairment.

## Results

The frequency of each symptom in the 5854 participants with HD with at least one endorsed symptom (positive or negative) varied from 10.8% (psychosis) to 66.0% (depression) and differed significantly by sex for depression, which was more common in women, and irritability and violent/aggressive behavior, which were both more common in men (Table 1). Symptoms were significantly (positively) correlated with each other (Supplemental Table S2), resulting in individuals exhibiting multiple symptoms.

**Table 1:** HD Symptom Counts and Sex Differences in the 5854 Individuals With at Least One Recorded HD Symptom Diagnosis (Positive or Negative)

Symptom	Freq, %	Male		Female		OR F vs. M (95% CI)	*p* Value (F vs. M)
		+	−	+	−		
Depression	66.0	1639	1120	2116	816	1.75 (1.57, 1.96)	<2 × 10^−16^
Irritability	60.3	1757	1005	1675	1252	0.77 (0.69, 0.85)	8.76 × 10^−7^
Psychosis	10.8	299	2396	301	2562	0.94 (0.79, 1.12)	.485
Violent/Aggressive Behavior	31.2	965	1794	802	2107	0.71 (0.63, 0.79)	1.90 × 10^−9^
Apathy	53.8	1484	1291	1581	1344	1.02 (0.92, 1.14)	.664
Perseverative/Obsessive Behavior	37.5	1048	1713	1076	1833	0.96 (0.86, 1.07)	.451
Cognitive Impairment	57.5	1583	1214	1726	1235	1.07 (0.97, 1.19)	.194

+/−, number of individuals of each sex with/without the symptom. Freq is the frequency of the symptom among individuals in the sample for whom a diagnosis of that symptom was recorded.

CI, confidence interval; F, female; Freq, frequency; HD, Huntington’s disease; M, male; OR, odds ratio.

We approximated disease duration as the age at the most recent observation available minus age at motor onset for REGISTRY and Enroll-HD individuals. Mean duration of HD was significantly lower in Enroll-HD, at 6.54 years, than REGISTRY, at 8.14 years (*p* = 6.55 × 10^−21^). The effects of CAG length, age at motor onset, sex, and disease duration on symptom presence were tested simultaneously via logistic regression (Table 2), along with potential differences in symptom frequency between REGISTRY versus Enroll-HD. Increased disease duration was significantly correlated with symptom presence for all symptoms except depression. These associations remained significant when age at most recent visit was included in the model (to account for increasing frequency of symptoms with increasing age) instead of age at motor onset (results not shown). Thus, the increased frequency of symptoms with disease duration cannot simply be attributed to age. Perseverative/obsessive behavior was seen substantially more frequently in Enroll-HD than REGISTRY participants (*p* = 5.72 × 10^−10^); otherwise, there were no significant differences.

**Table 2:** Effect of Sex, CAG, Age at Motor Onset, Disease Duration, and Sample Collection (Enroll-HD vs. REGISTRY) on HD Symptom Frequency

HD Symptom	Sex (F vs. M)		CAG		AMO		Duration		Enroll-HD vs. REGISTRY		AUC
	OR	*p* Value	OR	*p* Value	OR	*p* Value	OR	*p* Value	OR	*p* Value	
Depression	1.77^a^	8.44 × 10^−^^23^^a^	0.95^a^	7.64 × 10^−5^^a^	0.98^a^	6.35 × 10^−6^^a^	1.01	.0572	1.09	.198	0.592
Irritability	0.77^a^	2.36 × 10^−6^^a^	0.94^a^	1.06 × 10^−5^	0.98^a^	3.85 × 10^−9^^a^	1.02^a^	.000452^a^	1.06	.361	0.570
Psychosis	0.96	.684	1.02	.313	1.01	.334	1.06^a^	2.77 × 10^−14^^a^	0.88	.242	0.599
VAB	0.70^a^	1.04 × 10^−9^^a^	1.00	.863	0.99^a^	7.95 × 10^−5^^a^	1.04^a^	1.32 × 10^−12^^a^	0.97	.630	0.601
Apathy	1.04	.482	1.02	.156	1.00	.900	1.04^a^	9.84 × 10^−15^^a^	1.06	.411	0.572
POB	0.99	.865	1.02	.0603	1.00	.322	1.05^a^	1.28 × 10^−19^^a^	1.52^a^	5.72 × 10^−10^^a^	0.586
Cognitive Impairment	1.10	.0814	1.05^a^	8.75 × 10^−5^^a^	1.01	.112	1.10^a^	2.08 × 10^−54^^a^	0.96	.540	0.638

Analysis applied to 5854 individuals with at least one recorded symptom endorsement (not necessarily positive). All variables fitted simultaneously. AUC is a measure of the ability of the covariates to jointly predict symptom presence.

AMO, age at motor onset; AUC, area under the curve; F, female; HD, Huntington’s disease; M, male; OR, odds ratio; POB, perseverative/obsessive behavior; VAB, violent/aggressive behavior.

a Statistically significant association.

**Figure 1:**
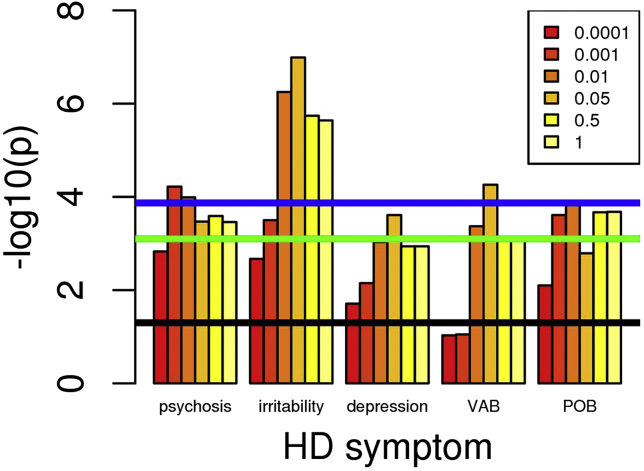
Association of increased schizophrenia polygenic risk score (PRS) with increased Huntington’s disease (HD) symptom frequency. Numbers in the box correspond to the *p*-value thresholds used to derive the PRS. Black line corresponds to nominally significant association (*p* = .05). Green line indicates associations significant after Bonferroni correction for 63 PRS-symptom comparisons (9 PRS × 7 symptoms). Blue line indicates associations significant after Bonferroni correction for 63 PRS-symptom comparisons and 6 PRS cutoffs. Note: Only symptoms with at least one *p* value reaching the green line are shown. POB, perseverative/obsessive behavior; VAB, violent/aggressive behavior.

**Figure 2:**
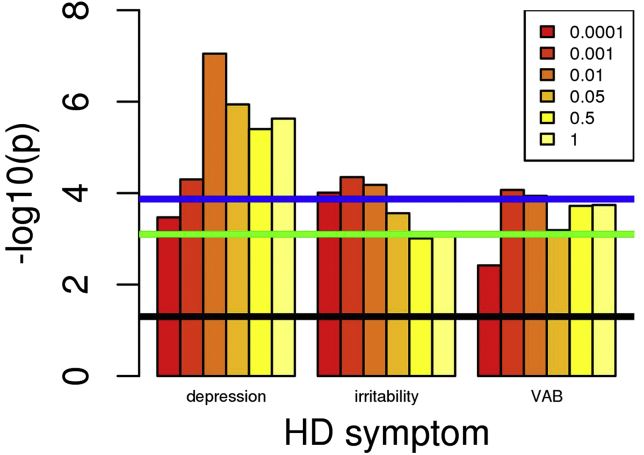
Association of increased major depression polygenic risk score (PRS) with increased Huntington’s disease (HD) symptom frequency. Numbers in the box correspond to the *p*-value thresholds used to derive the PRS. Black line corresponds to nominally significant association (*p* = .05). Green line indicates associations significant after Bonferroni correction for 63 PRS-symptom comparisons (9 PRS × 7 symptoms). Blue line indicates associations significant after Bonferroni correction for 63 PRS-symptom comparisons and 6 PRS cutoffs. Note: Only symptoms with at least one *p* value reaching the green line are shown. VAB, violent/aggressive behavior.

**Figure 3:**
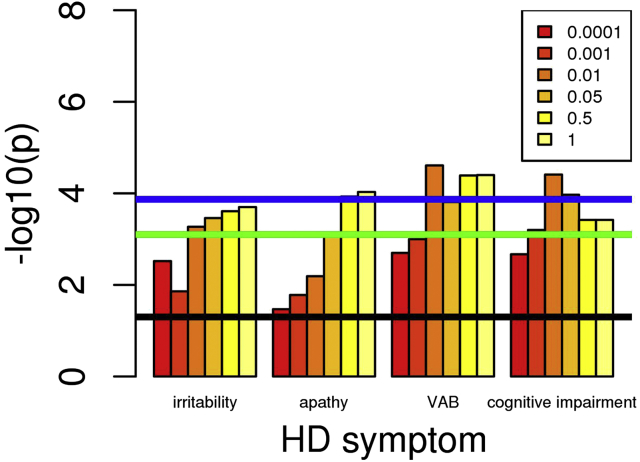
Association of decreased intelligence polygenic risk score (PRS) with increased Huntington’s disease (HD) symptom frequency. Numbers in the box correspond to the *p*-value thresholds used to derive the PRS. Black line corresponds to nominally significant association (*p* = .05). Green line indicates associations significant after Bonferroni correction for 63 PRS-symptom comparisons (9 PRS × 7 symptoms). Blue line indicates associations significant after Bonferroni correction for 63 PRS-symptom comparisons and 6 PRS cutoffs. Note: Only symptoms with at least one *p* value reaching the green line are shown. VAB, violent/aggressive behavior.

Supplemental Table S3 lists the PRS-symptom associations reaching nominal (*p* < .05) significance, with only the PRS cutoff giving the most significant result being shown. Full PRS-symptom analyses are shown in Supplemental Tables S4 to S12. The schizophrenia PRS showed significant associations with psychosis, irritability, and violent/aggressive behavior after correction for both the number of PRS-symptom tests and the 6 PRS cutoffs, as did the MDD PRS with depression, irritability, and violent/aggressive behavior. The associations of schizophrenia PRS with depression and perseverative/obsessive behavior were significant after correction for the number of PRS-symptom tests, as was the association between BPD PRS and depression. Of the primary hypotheses, the following nominally significant associations were also observed: MDD PRS with apathy, ADHD PRS with violent/aggressive behavior, PD PRS with cognitive impairment, and (interestingly, given the relatively small training GWAS) OCD PRS with perseverative/obsessive behavior. In each case, increased PRS was associated with an increased risk of the symptom (as shown by odds ratios > 1 in Supplemental Table S3). Associations of the intelligence PRS with violent/aggressive behavior, apathy, and cognitive impairment were significant after correction for both the number of PRS-symptom tests and the 6 PRS cutoffs, while the association between intelligence PRS and irritability was significant after correction for the number of PRS-symptom tests. The associations with intelligence PRS have odds ratio < 1 in Table 2, indicating that decreased PRS (i.e., reduced intelligence) is associated with increased risk of the symptom. In general, PRS-symptom associations in Supplemental Table S3 surviving correction for multiple testing of symptom-PRS comparisons showed at least nominally significant association over a range of PRS cutoffs (Figures 1, 2, and 3 and Supplemental Tables S4, S5, and S12), thus increasing confidence that these results are robust. Conversely, the association between PD PRS and cognitive impairment was only significant for PRS cutoff *p* < .0001 (Supplemental Table S11), making it likely that this is a false positive. This is also the case for the apparent association between psychosis and AD PRS (Supplemental Table S10). It can be seen from Supplemental Table S3 that the proportion of symptom variance accounted for by the PRS, as measured by the Nagelkerke *R*^2^, is small (<1%). Likewise, the ability of the PRS to predict symptom presence, as measured by the area under the curve (AUC), is limited (AUCs < .55). For comparison, the PGC (9) observed that schizophrenia PRS accounted for ∼7% of variance in schizophrenia liability, with AUC around 0.7. AUC of 0.8 is generally required for a clinically useful predictor (30).

Since sex, CAG length, age at motor onset, and disease duration were found to correlate significantly with symptom risk (Table 2), the association analyses between PRS and symptom were repeated conditioning on the effects of the factors found to be significantly associated with symptom presence. This made little difference to the significance of the PRS-symptom associations (Supplemental Table S13). Together, these factors give an AUC of 0.57 to 0.64 (Table 2), and adding the PRS makes little difference to this (Supplemental Table S13).

For symptoms with a significant association with sex (depression, irritability, and violent/aggressive behavior) and PRS with a significant association in the whole sample, the PRS-symptom association analyses were run in male and female subjects separately (Supplemental Table S14). There were no significant differences in odds ratio between male and female subjects.

PRS for schizophrenia, MDD, BPD, and intelligence are associated with multiple symptoms in Supplemental Table S3. Since these symptoms are correlated (Supplemental Table S2), it is unclear which symptom is driving the association. Likewise, symptoms are often associated with multiple PRS in Supplemental Table S3. These PRS will be correlated, owing to genetic overlap between psychiatric disorders (13). We therefore performed logistic regression of each symptom on all the significant PRS from Supplemental Table S3 simultaneously, including the 6 other symptom diagnoses as covariates (Table 3). This analysis highlighted specific associations between MDD PRS and depression, schizophrenia PRS and both psychosis and irritability, BPD PRS and violent/aggressive behavior, and intelligence PRS and cognitive decline. AD PRS and PD PRS were significantly associated with psychosis and cognitive impairment, respectively, but these are likely to be false positives, as discussed above.

**Table 3:** Logistic Regression of Symptom on Multiple PRS Simultaneously, Correcting for the Other Symptoms

Symptom	PRS	PRS Cutoff	OR	*p* Value
Depression	MDD^a^^,^^b^	0.01^a^	1.14^a^	8.52 × 10^−^^5^^a^
	Schizophrenia^b^	0.05	1.04	.253
	BPD^b^	0.01	1.07	.0617
	Intelligence	0.001	0.99	.679
Irritability	Schizophrenia^a^^,^^b^	0.05^a^	1.09^a^	.0164^a^
	MDD^b^	0.001	1.07	.117
	Intelligence	1	0.97	.384
Psychosis	Schizophrenia^a^^,^^b^	0.001^a^	1.14^a^	.00688^a^
	MDD	0.05	1.05	.302
	Alzheimer’s^a^	0.001^a^	1.13^a^	.0139^a^
	Intelligence	0.0001	0.95	.296
VAB	Intelligence	0.01	0.93	.0630
	Schizophrenia^b^	0.05	1.03	.480
	MDD	0.001	1.04	.272
	BPD^a^	0.001^a^	1.14^a^	9.11 × 10^−4^^a^
	ADHD^b^	0.05	1.05	.209
	ASD	0.01	1.04	.262
POB	Schizophrenia	0.01	1.03	.446
	BPD^b^	0.5	1.06	.0725
	Intelligence	0.01	0.97	.393
	MDD	0.5	1.01	.783
	OCD	0.001	1.07	.0532
Apathy	Intelligence	1	0.95	.137
	MDD^b^	0.05	1.00	.980
	BPD	0.001	1.05	.110
	Schizophrenia^b^	0.0001	1.03	.380
	ASD	0.001	1.06	.0808
Cognitive Impairment	Intelligence^a^^,^^b^	0.01^a^	0.91^a^	.00411^a^
	Parkinson’s^a^^,^^b^	0.0001^a^	1.10^a^	.00355^a^
	MDD	0.5	1.03	.319

Symptom-PRS pairs included in the analysis if they reach nominal significance (*p* < .05) when analyzed alone. The PRS cutoff giving the most significant association was used.

ADHD, attention-deficit/hyperactivity disorder; ASD, autism spectrum disorder; BPD, bipolar disorder; MDD, major depressive disorder; OCD, obsessive-compulsive disorder; OR, odds ratio; POB, perseverative/obsessive behavior; PRS, polygenic risk score; VAB, violent/aggressive behavior.

a Symptom-PRS pairs shown reach nominal significance (*p* < .05) after correction both for the other PRS shown in the table and for the other symptoms.

b Primary symptom-PRS hypotheses (see text).

The number of symptoms seen in any individual can be regarded as a surrogate for disease severity and might be expected to correlate with PRS. A linear regression of PRS on symptom count (0–7) was therefore performed (Supplemental Table S15). Significant associations with symptom count were observed for schizophrenia (*p* = 4.45 × 10^−9^), intelligence (*p* = 5.78 × 10^−8^), MDD (*p* = 4.17 × 10^−8^), and BD (*p* = .00268), with the latter being attributable to correlation with the other PRSs. To test whether association of PRS with individual symptoms can be explained by the general association with symptom count, the number of other symptoms was included as a covariate in the regression of PRS on symptom presence. Psychosis and irritability were found to be associated with schizophrenia PRS independently of other symptoms (Supplemental Table S16). Depression was associated with MDD PRS across all cutoffs (Supplemental Table S17), as was cognitive impairment with intelligence PRS (Supplemental Table S18). These results corroborate the specific PRS-symptom associations from the previous paragraph.

## Discussion

**Figure 4:**
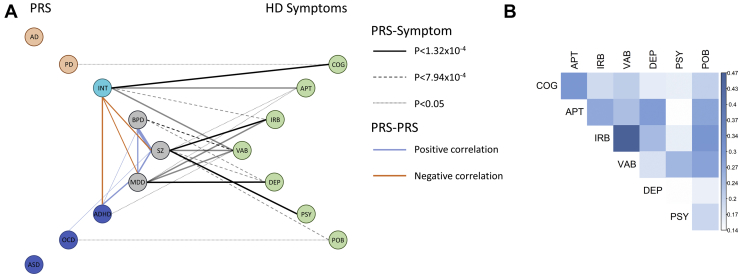
Pattern of association between Huntington’s disease (HD) symptoms and polygenic risk score (PRS) from psychiatric, neurodegenerative, and cognitive disorders. **(A)** PRS grouped into psychiatric (black), neurodevelopmental (blue), neurodegenerative (orange), and cognitive (cyan) disorders. Significant correlations between the PRSs associated with HD symptoms are shown by blue (positive correlations) and orange (negative correlations) lines, with line width proportional to the correlation magnitude. Solid lines between PRS and symptoms show associations significant after correcting for 63 PRS-symptom combinations (9 PRS × 7 symptoms) and 6 PRS cutoffs (*p* < 1.32 × 10^−4^). Dashed lines show associations significant after correcting for 63 PRS-symptom combinations (*p* < 7.94 × 10^−4^). Dotted lines show nominally significant associations (*p* < .05) in PRS-symptom combinations that were part of the primary analysis. Bold lines show PRS-symptom associations that are significant after correcting for other PRS and symptoms (Table 3). **(B)** Heat map showing correlations between symptoms (numerical values in Supplemental Table S2). All correlations are positive, and statistically significant (*p* < 2.2 × 10^−16^). AD, Alzheimer’s disease; ADHD, attention-deficit/hyperactivity disorder; APT, apathy; ASD, autism spectrum disorder; BPD, bipolar disorder; COG, cognitive impairment; DEP, depression; INT, intelligence; IRB, irritability; MDD, major depressive disorder; OCD, obsessive-compulsive disorder; PD, Parkinson’s disease; POB, perseverative/obsessive behavior; PSY, psychosis; SZ, schizophrenia; VAB, violent/aggressive behavior.

We show significant genetic overlaps between psychiatric disorders and psychiatric symptoms in HD, along with genetic overlap between intelligence and cognitive symptoms in HD. There is little overlap between the two neurodegenerative disorders and neuropsychiatric symptoms or cognition in HD. While the overall pattern of individual associations is complex, reflecting the genetic relationships between the disorders (see Figure 4 for a graphical overview), we were able to identify that MDD PRS is specifically associated with depression, schizophrenia PRS with psychosis and irritability, BPD PRS with violent/aggressive behavior, and intelligence PRS with cognitive impairment.

Our results are consistent with observations of shared genetic risk among psychiatric disorders but no overlap of genetic risk between the individual neurological disorders and no overlap of genetic risk for neurological disorders with neuropsychiatric symptom risk in neurodegenerative disorders (13,21,29). By contrast, common genetic variation is associated with behavioral phenotypes (such as autistic behavior and developmental delay) in severe monogenic neurodevelopmental disorders, with similar effect sizes (*R*^2^ of 0.6%–0.8%) to those observed here (31). These results, along with ours, indicate that even in diseases previously assumed to be entirely attributable to single genetic variants, there is a contribution of polygenic risk in influencing phenotypic presentation (31).

Schizophrenia PRS is associated with psychosis (and irritability) independently of other symptoms and is the only PRS to predict HD psychosis. While psychosis in HD is around 10 times more common than in the general population, it is seen in only a minority of cases (2), as in the participants studied here (11%). There are also reports of clustering of psychotic symptoms in HD families (6,7), implying a genetic contribution to these symptoms. Previously, Tsuang *et al.* (8) tested for association of psychosis in HD with a set of 214 SNPs chosen from candidate genes for schizophrenia, HD, or psychosis in neurodegenerative disorders. None of their associations survived correction for multiple testing, possibly owing to the small sample size (47 cases with HD with psychosis, 126 without psychosis). Of the SNPs tested by Tsuang *et al.*, 183 were available in our data; a PRS generated from these (with effect sizes taken from the PGC schizophrenia GWAS) showed no association with psychosis in our HD sample (odds ratio = 0.97, *p* = .536). The highly significant association between schizophrenia PRS and psychosis observed in our sample shows the benefits of a large sample size of patients with HD, along with a larger set of SNPs systematically selected for association with schizophrenia in a powerful schizophrenia GWAS. The schizophrenia PRS is also the most significantly associated with perseveration, one of the most characteristic behavioral problems in HD. The PRS for OCD also influences the presence of perseverative/obsessive behavior (Table 3, Supplemental Table S3), despite the OCD PRS having been derived from a much smaller and less powerful sample than that for schizophrenia.

Schizophrenia may be regarded as a neurodevelopmental disorder with origins in fetal development (32) that manifest in symptoms most usually in early adulthood, whereas psychotic symptoms in HD generally manifest later in life (2,33). Genes expressed in the medium spiny neurons of the striatum are among the neuronal subtypes most enriched for schizophrenia risk (34). Medium spiny neurons are the most vulnerable cell types in HD: by the time HD motor symptoms are manifest, up to half of these neurons have died (35). Susceptibility to psychiatric symptoms in surviving cells may therefore be influenced by the schizophrenia polygenic risk, providing a hypothesis for the increased rates of psychotic symptoms observed in patients with HD.

Cognitive decline and dementia are part of HD progression, with executive and psychomotor function often the first deficits noted and with memory problems later in disease (2). Longer CAG repeat tracts are associated with increased risk of cognitive deficits in our sample: the previous data on this relationship have been inconsistent (2,36). Recent studies have shown that poorer performance in the symbol digit modality test was associated with longer *HTT* CAG repeat lengths (37), and poorer cognitive function was seen with increasing repeat lengths in the disease-causing range (38). The clinical characteristics questionnaire used to assess symptoms for this study seeks an integrated measure of cognitive decline by asking about problems that might interfere with performing everyday functions and is relatively crude, although this will be partly offset by the larger size of our study (thousands rather than hundreds of participants).

It is notable that neither apathy nor cognitive dysfunction in HD is correlated with PRS for psychiatric or neurodegenerative disorders apart from nominal associations between apathy and MDD and between schizophrenia and BPD PRS, which become nonsignificant when accounting for the presence of other symptoms (see Table 3). However, both are significantly correlated with a PRS measuring intelligence. This suggests that apathy in HD should be considered as a cognitive, rather than psychiatric, symptom (unlike depression) and is also consistent with apathy being the only psychiatric symptom to correlate with disease progression (39). Higher PRS for intelligence is associated with later cognitive decline in HD, as it is in AD (14). The genes associated with intelligence are highly expressed in medium spiny neurons of the striatum and pyramidal neurons of the CA1 hippocampus (14), early targets of degeneration in HD and AD, respectively, potentially contributing to the severe cognitive decline seen in these diseases. Surprisingly, there is no significant association between the presence of cognitive deficits in HD and genetic risk for AD, although AD PRS predicts memory decline and poorer cognitive performance in healthy children and adults well before the age of risk for AD (40,41). In children, *HTT* CAG repeat length itself shows an inverted J-shaped relationship with cognition, with maximum cognition at 40 to 41 repeats (38). Since higher intelligence is associated with better health and increased well-being (42), there may thus be a selective advantage of longer *HTT* CAG repeat length, below the threshold for HD, in the wider population.

Depression is very common in HD and more common in females (2). MDD PRS is specifically associated with increased risk of depression in HD (Table 3). However, there were no significant sex differences in these associations (Supplemental Table S14). There is a significant correlation in our sample of increased likelihood of depression with shorter CAG repeat lengths in the disease-causing allele in *HTT* (Table 2), along with an association with earlier age at motor onset. The reason for these associations is unclear; the obvious explanation that longer disease duration makes depression more likely is not the case in our sample (Table 2). In fact, the apparent associations between depression and both age at motor onset and CAG repeat length are explained by the residual of age at motor onset after correction for CAG length [see Lee *et al.* (5) for how this is derived]. Participants with an earlier than expected age at onset (given their CAG length) are more likely to have depression. This could be due to increased environmental stress among individuals with earlier motor onset and also because some genetic modifiers of age at motor onset may also be associated with depression in HD. The few previous studies, each examining fewer than 100 participants, detected no relationship between CAG length and presence of depression (43, 44, 45). In the non-disease-causing range, longer *HTT* CAG length from 24 to 38 repeats was associated with an increased likelihood of depression (46).

Irritability, like depression, is very common in HD and has a significantly reduced risk with longer CAG length (Table 2), but unlike depression, longer duration of disease makes irritability more likely. This association is not explained by increased age. Schizophrenia PRS is associated with increased risk of irritability (Table 3, Supplemental Table S3). Violent/aggressive behavior can be considered to be an extreme manifestation of irritability and is associated with PRS for BPD (Table 3), with the apparently more significant associations with schizophrenia, intelligence, and MDD PRS (Supplemental Table S3) attributable to correlations between violent/aggressive behavior and other symptoms. As in the wider population, violence and aggressive behavior are more common in men (2).

The study presented here has a number of limitations. As noted, the clinical characteristics questionnaire is a relatively crude instrument. The larger size of this study compared with previous studies partly mitigates its limitations, and these initial findings provide a platform for more detailed studies using specific psychiatric instruments. We note that all Enroll-HD participants now undergo a short problem behaviors assessment battery, which may account for the increased frequency of perseverative/obsessive behavior observed in Enroll-HD relative to REGISTRY. It would be useful to investigate further the predictive power of the PRS using more detailed clinical data, including age at onset, on the psychiatric symptoms in HD. It would also be interesting to explore the overlap of multiple symptoms and the psychiatric and cognitive PRS to attempt to establish directions of causation.

It is notable that the PRSs for psychiatric diseases are associated with increased risk of developing parallel phenotypic behavioral and psychiatric symptoms in participants with HD. The data available in the ongoing Enroll-HD study will provide for much more detailed analyses of these symptoms and their etiology and treatment and may in turn inform the study of psychiatric diseases. The lack of genetic overlap with other neurodegenerative disorders, consistent with the Brainstorm study (13), implies different underlying pathways leading to degeneration in the different neurodegenerations that may relate to the disease-specific characteristic differential neuronal vulnerabilities to degeneration. Striatal medium spiny neurons are most vulnerable in HD, dopaminergic neurons of the substantia nigra are most vulnerable in PD, and hippocampal and entorhinal cortical neurons are most vulnerable in AD. The differential vulnerability may relate to pathways essential to the survival and continued function of each specific cell type, and these are likely to be different in different neurodegenerations (47). Thus, psychiatric symptoms may be mediated by common dysfunctional pathways in surviving cells, whereas cognitive and neurodegenerative symptoms are likely due to specific regional cellular populations degenerating via different pathways. Further study of the relevant genes and pathways may provide a rationale for the application of treatments used in the wider population for depression and psychosis to these symptoms in HD.

## Supplementary Material

Supplemental Material

Key Resources Table

## References

[1] Bates G.P., Dorsey R., Gusella J.F., Hayden M.R., Kay C., Leavitt B.R. (2015). Huntington disease. Nat Rev Dis Prim.

[2] Craufurd D., Snowden J., Bates G.P., Tabrizi S.J., Jones L. (2014). Neuropsychiatry and neuropsychology. Huntington’s Disease.

[3] Huntington’s GM of, Disease Consortium (GeM-HD) (2015). Identification of genetic factors that modify clinical onset of Huntington’s disease. Cell.

[4] Holmans P.A., Massey T.H., Jones L. (2017). Genetic modifiers of Mendelian disease: Huntington’s disease and the trinucleotide repeat disorders. Hum Mol Genet.

[5] Lee J.-M., Correia K., Loupe J., Kim K.-H., Barker D., Hong E.P. (2019). Huntington’s disease onset is determined by length of uninterrupted CAG, not encoded polyglutamine, and is modified by DNA maintenance mechanisms. bioRxiv.

[6] Lovestone S., Hodgson S., Sham P., Differ A.M., Levy R. (1996). Familial psychiatric presentation of Huntington’s disease. J Med Genet.

[7] Tsuang D., Almqvist E.W., Lipe H., Strgar F., DiGiacomo L., Hoff D. (2000). Familial aggregation of psychotic symptoms in Huntington’s disease. Am J Psychiatry.

[8] Tsuang D.W., Greenwood T.A., Jayadev S., Davis M., Shutes-David A., Bird T.D. (2018). A genetic study of psychosis in Huntington’s disease: Evidence for the involvement of glutamate signaling pathways. J Huntingtons Dis.

[9] Schizophrenia Working Group of the Psychiatric Geonomics Consortium (2014). Biological insights from 108 schizophrenia-associated genetic loci. Nature.

[10] Stahl E.A., Breen G., Forstner A.J., McQuillin A., Ripke S., Trubetskoy V. (2019). Genome-wide association study identifies 30 loci associated with bipolar disorder. Nat Genet.

[11] Howard D.M., Adams M.J., Clarke T.K., Hafferty J.D., Gibson J., Shirali M. (2019). Genome-wide meta-analysis of depression identifies 102 independent variants and highlights the importance of the prefrontal brain regions. Nat Neurosci.

[12] Demontis D., Walters R.K., Martin J., Mattheisen M., Als T.D., Agerbo E. (2019). Discovery of the first genome-wide significant risk loci for attention deficit/hyperactivity disorder. Nat Genet.

[13] Anttila V., Bulik-Sullivan B., Finucane H.K., Walters R.K., Bras J., Duncan L. (2018). Analysis of shared heritability in common disorders of the brain. Science.

[14] Savage J.E., Jansen P.R., Stringer S., Watanabe K., Bryois J., de Leeuw C.A. (2018). Genome-wide association meta-analysis in 269,867 individuals identifies new genetic and functional links to intelligence. Nat Genet.

[15] Murray P.S., Kumar S., Demichele-Sweet M.A.A., Sweet R.A. (2014). Psychosis in Alzheimer’s disease. Biol Psychiatry.

[16] Barrett M.J., Smolkin M.E., Flanigan J.L., Shah B.B., Harrison M.B., Sperling S.A. (2017). Characteristics, correlates, and assessment of psychosis in Parkinson disease without dementia. Parkinsonism Relat Disord.

[17] Ravina B., Marder K., Fernandez H.H., Friedman J.H., McDonald W., Murphy D. (2007). Diagnostic criteria for psychosis in Parkinson’s disease: Report of an NINDS, NIMH work group. Mov Disord.

[18] McKeith I.G., Boeve B.F., Dickson D.W., Halliday G., Taylor J.-P., Weintraub D. (2017). Diagnosis and management of dementia with Lewy bodies: Fourth Concensus Report of the DLB Consortium. Neurology.

[19] Hollingworth P., Hamshere M.L., Holmans P.A., O’Donovan M.C., Sims R., Powell J. (2007). Increased familial risk and genomewide significant linkage for Alzheimer’s disease with psychosis. Am J Med Genet B Neuropsychiatr Genet.

[20] Sweet R.A., Bennett D.A., Graff-Radford N.R., Mayeux R. (2010). Assessment and familial aggregation of psychosis in Alzheimer’s disease from the National Institute on Aging Late Onset Alzheimer’s Disease Family Study. Brain.

[21] DeMichele-Sweet M.A.A., Weamer E.A., Klei L., Vrana D.T., Hollingshead D.J., Seltman H.J. (2018). Genetic risk for schizophrenia and psychosis in Alzheimer disease. Mol Psychiatry.

[22] Orth M., Handley O.J., Schwenke C., Dunnett S.B., Craufurd D., Ho A.K. (2010). Observing Huntington’s disease: The European Huntington’s Disease Network’s REGISTRY. PLoS Curr.

[23] Walker T., Ghosh B., Kipps C. (2017). Assessing decline: Visualising progression in Huntington’s disease using a clinical dashboard with Enroll-HD data. J Huntingtons Dis.

[24] Purcell S.M., Wray N.R., Stone J.L., Visscher P.M., O’Donovan M.C., International Schizophrenia Consortium (2009). Common polygenic variation contributes to risk of schizophrenia and bipolar disorder. Nature.

[25] Chang C.C., Chow C.C., Tellier L.C.A.M., Vattikuti S., Purcell S.M., Lee J.J. (2015). Second-generation PLINK: Rising to the challenge of larger and richer datasets. Gigascience.

[26] Grove J., Ripke S., Als T.D., Mattheisen M., Walters R.K., Won H. (2019). Identification of common genetic risk variants for autism spectrum disorder. Nat Genet.

[27] Arnold P.D., Askland K.D., Barlassina C., Bellodi L., Bienvenu O.J., (OCGAS) IOCDFGC (IOCDF-G and OCDCGAS (2017). Revealing the complex genetic architecture of obsessive–compulsive disorder using meta-analysis. Mol Psychiatry.

[28] Lambert J.C., Ibrahim-Verbaas C.A., Harold D., Naj A.C., Sims R., Bellenguez C. (2013). Meta-analysis of 74,046 individuals identifies 11 new susceptibility loci for Alzheimer’s disease. Nat Genet.

[29] Nalls M.A., Blauwendraat C., Vallerga C.L., Heilbron K., Bandres-Ciga S., Chang D. (2019). Identification of novel risk loci, causal insights, and heritable risk for Parkinson’s disease: A meta-analysis of genome-wide association studies. Lancet Neurol.

[30] Schummers L., Himes K.P., Bodnar L.M., Hutcheon J.A. (2016). Predictor characteristics necessary for building a clinically useful risk prediction model: A simulation study. BMC Med Res Methodol.

[31] Niemi M.E.K., Martin H.C., Rice D.L., Gallone G., Gordon S., Kelemen M. (2018). Common genetic variants contribute to risk of rare severe neurodevelopmental disorders. Nature.

[32] Clifton N.E., Hannon E., Harwood J.C., Di Florio A., Thomas K.L., Holmans P.A. (2019). Dynamic expression of genes associated with schizophrenia and bipolar disorder across development. Transl Psychiatry.

[33] Eddy C.M., Parkinson E.G., Rickards H.E. (2016). Changes in mental state and behaviour in Huntington’s disease. Lancet Psychiatry.

[34] Skene N.G., Bryois J., Bakken T.E., Breen G., Crowley J.J., Gaspar H.A. (2018). Genetic identification of brain cell types underlying schizophrenia. Nat Genet.

[35] Vonsattel J.P., DiFiglia M. (1998). Huntington disease. J Neuropathol Exp Neurol.

[36] Podvin S., Reardon H.T., Yin K., Mosier C., Hook V. (2019). Multiple clinical features of Huntington’s disease correlate with mutant HTT gene CAG repeat lengths and neurodegeneration. J Neurol.

[37] Tabrizi S.J., Scahill R.I., Owen G., Durr A., Leavitt B.R., Roos R.A. (2013). Predictors of phenotypic progression and disease onset in premanifest and early-stage Huntington’s disease in the TRACK-HD study: Analysis of 36-month observational data. Lancet Neurol.

[38] Lee J.K., Conrad A., Epping E., Mathews K., Magnotta V., Dawson J.D., Nopoulos P. (2018). Effect of trinucleotide repeats in the Huntington’s gene on intelligence. EBioMedicine.

[39] Thompson J.C., Harris J., Sollom A.C., Stopford C.L., Howard E., Snowden J.S., Craufurd D. (2012). Longitudinal evaluation of neuropsychiatric symptoms in Huntington’s disease. J Neuropsychiatry Clin Neurosci.

[40] Marden J.R., Mayeda E.R., Walter S., Vivot A., Tchetgen Tchetgen E.J., Kawachi I., Glymour M.M. (2016). Using an Alzheimer disease polygenic risk score to predict memory decline in black and white Americans over 14 years of follow-up. Alzheimer Dis Assoc Disord.

[41] Axelrud L.K., Santoro M.L., Pine D.S., Talarico F., Gadelha A., Manfro G.G. (2018). Polygenic risk score for Alzheimer’s disease: Implications for memory performance and hippocampal volumes in early life. Am J Psychiatry.

[42] Wraw C., Deary I.J., Gale C.R., Der G. (2015). Intelligence in youth and health at age 50. Intelligence.

[43] Weigell-Weber M., Schmid W., Spiegel R. (1996). Psychiatric symptoms and CAG expansion in Huntington’s disease. Am J Med Genet.

[44] Zappacosta B., Monza D., Meoni C., Austoni L., Soliveri P., Gellera C. (1996). Psychiatric symptoms do not correlate with cognitive decline, motor symptoms, or CAG repeat length in Huntington’s disease. Arch Neurol.

[45] Berrios G.E., Wagle A.C., Markova I.S., Wagle S.A., Ho L.W., Rubinsztein D.C. (2001). Psychiatric symptoms and CAG repeats in neurologically asymptomatic Huntington’s disease gene carriers. Psychiatry Res.

[46] Gardiner S.L., van Belzen M.J., Boogaard M.W., van Roon-Mom W.M.C., Rozing M.P., van Hemert A.M. (2017). Huntingtin gene repeat size variations affect risk of lifetime depression. Transl Psychiatry.

[47] Fu H., Hardy J., Duff K.E. (2018). Selective vulnerability in neurodegenerative diseases. Nat Neurosci.

