# Supplementary material for: Genetic Risk Underlying Psychiatric and Cognitive Symptoms in Huntington’s Disease
Source: Biol Psychiatry. 2020 May 1;87(9):857–65. doi: 10.1016/j.biopsych.2019.12.010 (PMC7156911; doi:10.1016/j.biopsych.2019.12.010)
Supplement: Supplemental Material [file mmc1.pdf]

Genetic Risk Underlying Psychiatric and Cognitive Symptoms in Huntington's Disease

Supplementary Information

Supplementary Figure S1:

Flow diagram showing how the sample used to test association between PRS and symptoms was derived.

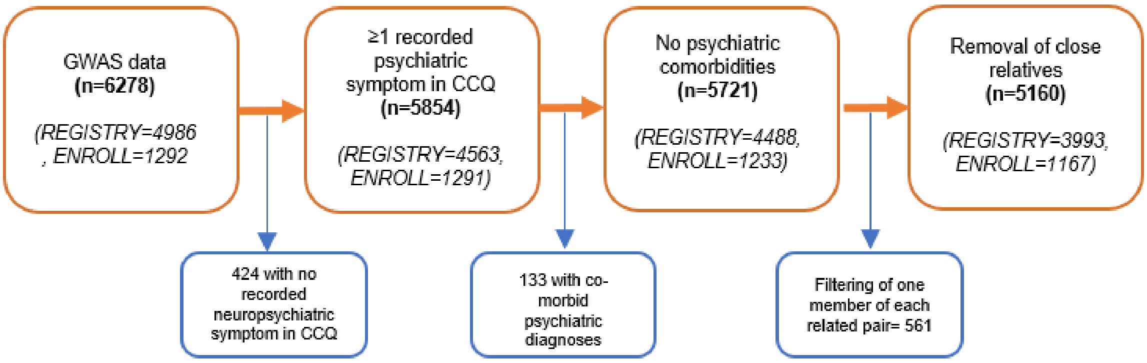

Supplementary Table S1: Sample sizes of GWAS used as training sets for polygenic risk scores.

DISORDER	NUMBER OF CASES	NUMBER OF CONTROLS
Major depressive disorder (MDD)	170,756	329,443
Bipolar disorder (BPD)	20,352	31,358
Schizophrenia (SCZ)	36,989	113,075
Attention deficit hyperactivity disorder (ADHD)	20,183	35,191
Autism spectrum disorder (ASD)	18,382	27,969
Obsessive-compulsive disorder (OCD)	2,688	7,037
Alzheimer's Disease (AD)	17,008	37,154
Parkinson's Disease (PD)	26,421	442,271
Intelligence	269,867 individuals	

Supplementary Table S2: Correlations between symptoms.

	irb	psy	VAB	cog	apt	POB
dep	0.242	0.139	0.184	0.168	0.291	0.161
irb		0.162	0.466	0.194	0.270	0.303
psy			0.250	0.165	0.139	0.199
VAB				0.213	0.234	0.281
cog					0.300	0.210
apt						0.278

All correlations highly significant ($p<2.2x10^{-16}$)

dep = depression, irb = irritability, psy = psychosis, VAB = violent/aggressive behaviour. POB= perseverative/obsessive behaviour. cog=cognitive impairment, apt=apathy

Supplementary Table S3: Individual HD symptom-polygenic risk score associations reaching nominal significance

SYMPTOM	Risk score	PRS CUT-OFF	OR	R^2 (%)	AUC	P VALUE
Depression	**MDD**	**0.01**	**1.17**	**0.78**	**0.547**	**9.01×10^{-8}**
	Schizophrenia	*0.05*	*1.12*	*0.37*	*0.528*	*2.47×10^{-4}*
	BPD	*0.01*	*1.11*	*0.36*	*0.532*	*3.06×10^{-4}*
	Intelligence	0.001	0.93	0.18	0.519	9.51×10^{-3}
Irritability	**Schizophrenia**	**0.05**	**1.17**	**0.76**	**0.542**	**1.03×10^{-7}**
	**MDD**	**0.001**	**1.12**	**0.45**	**0.535**	**4.42×10^{-5}**
	Intelligence	*1*	*0.90*	*0.37*	*0.531*	*1.99×10^{-4}*
Psychosis	**Schizophrenia**	**0.001**	**1.20**	**0.64**	**0.548**	**5.98×10^{-5}**
	MDD	0.05	1.14	0.34	0.537	3.40×10^{-3}
	Alzheimer’s	0.001	1.10	0.19	0.529	0.0265
	Intelligence	0.0001	0.90	0.24	0.534	0.0147
VAB	**Intelligence**	**0.01**	**0.89**	**0.50**	**0.537**	**2.44×10^{-5}**
	**Schizophrenia**	**0.05**	**1.13**	**0.46**	**0.532**	**5.46×10^{-5}**
	**MDD**	**0.001**	**1.13**	**0.44**	**0.536**	**8.45×10^{-5}**
	BPD	*0.001*	*1.11*	*0.34*	*0.527*	*5.35×10^{-4}*
	ADHD	0.05	1.09	0.22	0.523	5.26×10^{-3}
	ASD	0.01	1.06	0.11	0.517	0.0451
POB	*Schizophrenia*	*0.01*	*1.12*	*0.39*	*0.531*	*1.47×10^{-4}*
	BPD	0.5	1.08	0.18	0.521	9.25×10^{-3}
	Intelligence	0.01	0.93	0.18	0.521	0.0103
	MDD	0.5	1.07	0.15	0.519	0.0188
	OCD	0.001	1.07	0.15	0.521	0.0190
Apathy	**Intelligence**	**1**	**0.90**	**0.41**	**0.534**	**9.27×10^{-5}**
	MDD	0.05	1.08	0.18	0.521	9.17×10^{-3}
	BPD	0.001	1.07	0.14	0.519	0.0222
	Schizophrenia	0.0001	1.07	0.13	0.518	0.0254
	ASD	0.001	1.06	0.10	0.516	0.0499
Cognitive impairment	**Intelligence**	**0.01**	**0.89**	**0.45**	**0.535**	**3.90×10^{-5}**
	Parkinson's	0.0001	1.09	0.26	0.524	1.67×10^{-3}
	MDD	0.5	1.09	0.23	0.524	3.51×10^{-3}

VAB= violent/aggressive behaviour, POB=perserverative/obsessive behaviour. MDD= major depressive disorder. BPD= bipolar disorder. ASD= autism spectrum disorder. ADHD = attention deficit hyperactivity disorder. R^2= Nagelkerke R^2. OR= Odds ratio for presence of symptom associated with 1 s.d. increase in PRS.

Results in *italics* satisfy the Bonferroni corrected p-value cut-off 7.94×10^{-4} (corrected for 63 possible PRS-phenotype comparisons), and results in **bold** satisfy the Bonferroni corrected p-value cut of 1.32×10^{-4} (corrected for the 63 PRS-symptom comparisons and 6 PRS cut-offs). Primary symptom-PRS hypotheses (see text) are underlined.

Supplementary Table S4: All PRS-symptom analyses for SCZ PRS (no MHC)

Symptom	Statistic	PRS cutoff					
		0.0001	0.001	0.01	0.05	0.5	1
dep	OR	1.07(1.01,1.14)	1.08(1.02,1.15)	1.10(1.04,1.17)	1.12(1.05,1.18)	1.10(1.04,1.17)	1.10(1.04,1.17)
	R2	0.0015	0.0020	0.0030	0.0037	0.0029	0.0029
	p	0.0193	0.00715	0.000929	0.000247	0.00114	0.00115
irb	OR	1.09(1.03,1.16)	1.11(1.05,1.17)	1.16(1.09,1.22)	1.17(1.10,1.24)	1.15(1.09,1.22)	1.15(1.08,1.21)
	R2	0.0025	0.0035	0.0067	0.0076	0.0061	0.0060
	p	0.00213	0.000318	5.60e-7	1.03e-7	1.82e-6	2.31e-6
psy	OR	1.18(1.09,1.29)	1.20(1.10,1.31)	1.19(1.09,1.30)	1.17(1.08,1.28)	1.18(1.08,1.29)	1.17(1.08,1.28)
	R2	0.0057	0.0064	0.0060	0.0051	0.0053	0.0051
	p	0.000148	5.98e-5	0.000103	0.000342	0.000260	0.000350
apt	OR	1.07(1.01,1.13)	1.05(0.99,1.11)	1.06(1.00,1.12)	1.05(0.99,1.11)	1.03(0.98,1.09)	1.03(0.98,1.09)
	R2	0.0013	0.0008	0.0010	0.0007	0.0004	0.0004
	p	0.0254	0.0858	0.0549	0.0936	0.231	0.245
VAB	OR	1.05(0.99,1.12)	1.05(0.99,1.12)	1.11(1.05,1.18)	1.13(1.07,1.20)	1.11(1.04,1.18)	1.11(1.04,1.18)
	R2	0.0008	0.0008	0.0035	0.0046	0.0032	0.0032
	p	0.0927	0.0892	0.000432	5.46e-5	0.000807	0.000750
POB	OR	1.08(1.02,1.14)	1.11(1.05,1.18)	1.12(1.06,1.18)	1.10(1.04,1.16)	1.12(1.05,1.18)	1.12(1.05,1.18)
	R2	0.0019	0.0037	0.0039	0.0027	0.0037	0.0038
	p	0.00799	0.000246	0.000147	0.00161	0.000215	0.000208
cog	OR	1.01(0.95,1.07)	1.03(0.97,1.08)	1.05(1.00,1.11)	1.06(1.00,1.12)	1.04(0.98,1.10)	1.04(0.98,1.10)
	R2	2.87e-5	0.0002	0.0009	0.0009	0.0005	0.0005
	p	0.742	0.384	0.0678	0.0592	0.164	0.152

dep = depression, irb = irritability, psy = psychosis, apt = apathy, VAB = violent/aggressive behaviour. POB= perseverative/obsessive behaviour. cog=cognitive impairment.

Supplementary Table S5: All PRS-symptom analyses for MDD PRS

Symptom	Statistic	PRS cutoff					
		0.0001	0.001	0.01	0.05	0.5	1
dep	OR	1.11(1.05,1.18)	1.13(1.06,1.19)	1.17(1.11,1.24)	1.16(1.09,1.22)	1.15(1.08,1.22)	1.15(1.09,1.22)
	R2	0.0035	0.0045	0.0078	0.0064	0.0058	0.0061
	p	0.000339	5.04e-5	9.01e-8	1.14e-6	3.97e-6	2.34e-6
irb	OR	1.12(1.06,1.18)	1.12(1.06,1.19)	1.12(1.06,1.19)	1.11(1.05,1.17)	1.10(1.04,1.16)	1.10(1.04,1.16)
	R2	0.0041	0.0045	0.0043	0.0036	0.0029	0.0030
	p	9.85e-5	4.42e-5	6.67e-5	0.000274	0.000985	0.000823
psy	OR	1.08(0.99,1.18)	1.13(1.03,1.23)	1.13(1.03,1.23)	1.14(1.04,1.24)	1.12(1.03,1.22)	1.11(1.02,1.21)
	R2	0.0012	0.0028	0.0029	0.0034	0.0026	0.0022
	p	0.0785	0.00744	0.00655	0.00340	0.0109	0.0162
apt	OR	1.02(0.96,1.07)	1.04(0.98,1.10)	1.06(1.00,1.12)	1.08(1.02,1.14)	1.07(1.01,1.13)	1.07(1.01,1.13)
	R2	7.81e-5	0.0005	0.0012	0.0018	0.0016	0.0016
	p	0.588	0.160	0.0356	0.00917	0.0157	0.0149
VAB	OR	1.09(1.03,1.16)	1.13(1.06,1.20)	1.13(1.06,1.20)	1.11(1.05,1.18)	1.12(1.06,1.19)	1.12(1.06,1.19)
	R2	0.0023	0.0044	0.0042	0.0033	0.0039	0.0040
	p	0.00379	8.45e-5	0.000116	0.000652	0.000189	0.000183
POB	OR	1.00(0.95,1.06)	1.02(0.97,1.08)	1.05(0.99,1.11)	1.06(1.00,1.12)	1.07(1.01,1.13)	1.07(1.01,1.13)
	R2	7.51e-6	0.0002	0.0007	0.0010	0.0015	0.0013
	p	0.868	0.400	0.106	0.0570	0.0188	0.0275
cog	OR	1.02(0.96,1.07)	1.07(1.01,1.13)	1.05(0.99,1.11)	1.09(1.03,1.15)	1.09(1.03,1.15)	1.08(1.02,1.14)
	R2	8.72e-5	0.0015	0.0008	0.0023	0.0023	0.0020
	p	0.566	0.0167	0.0777	0.00354	0.00351	0.00546

dep = depression, irb = irritability, psy = psychosis, apt = apathy, VAB = violent/aggressive behaviour. POB= perseverative/obsessive behaviour. cog=cognitive impairment.

Supplementary Table S6: All PRS-symptom analyses for BPD PRS

Symptom	Statistic	PRS cutoff					
		0.0001	0.001	0.01	0.05	0.5	1
dep	OR	1.04(0.98,1.10)	1.07(1.00,1.13)	1.11(1.05,1.18)	1.11(1.05,1.18)	1.07(1.01,1.14)	1.07(1.01,1.14)
	R2	0.0005	0.0012	0.0036	0.0034	0.0016	0.0016
	p	0.198	0.0336	0.000306	0.000466	0.0161	0.0170
irb	OR	1.04(0.99,1.10)	1.04(0.98,1.10)	1.04(0.98,1.10)	1.01(0.96,1.07)	1.01(0.96,1.07)	1.01(0.96,1.07)
	R2	0.0006	0.0006	0.0005	4.20e-5	4.24e-5	4.11e-5
	p	0.138	0.150	0.161	0.692	0.691	0.695
psy	OR	1.00(0.91,1.09)	1.02(0.94,1.12)	1.05(0.97,1.15)	1.06(0.97,1.15)	1.05(0.96,1.15)	1.07(0.98,1.17)
	R2	1.72e-6	0.0001	0.0006	0.0006	0.0005	0.0009
	p	0.947	0.615	0.236	0.212	0.258	0.141
apt	OR	1.05(0.99,1.11)	1.07(1.01,1.13)	1.04(0.98,1.10)	1.00(0.94,1.05)	0.97(0.92,1.03)	0.98(0.92,1.03)
	R2	0.0006	0.0014	0.0005	3.44e-6	0.0002	0.0002
	p	0.120	0.0222	0.185	0.909	0.358	0.436
VAB	OR	1.06(1.00,1.13)	1.11(1.05,1.18)	1.08(1.01,1.14)	1.07(1.01,1.14)	1.08(1.01,1.14)	1.08(1.02,1.15)
	R2	0.0012	0.0034	0.0017	0.0014	0.0016	0.0019
	p	0.0413	0.000535	0.0152	0.0245	0.0175	0.00975
POB	OR	1.05(0.99,1.11)	1.05(0.99,1.11)	1.06(1.00,1.12)	1.07(1.01,1.13)	1.08(1.02,1.14)	1.08(1.02,1.14)
	R2	0.0008	0.0007	0.0010	0.0014	0.0018	0.0018
	p	0.0839	0.118	0.0553	0.0254	0.00925	0.0113
cog	OR	0.98(0.92,1.03)	1.00(0.95,1.06)	1.02(0.97,1.08)	1.03(0.97,1.09)	1.03(0.98,1.09)	1.03(0.97,1.09)
	R2	0.0002	3.76e-7	0.0002	0.0003	0.0003	0.0003
	p	0.421	0.970	0.451	0.305	0.275	0.323

dep = depression, irb = irritability, psy = psychosis, apt = apathy, VAB = violent/aggressive behaviour. POB= perseverative/obsessive behaviour. cog=cognitive impairment.

Supplementary Table S7: All PRS-symptom analyses for ADHD PRS

Symptom	Statistic	PRS cutoff					
		0.0001	0.001	0.01	0.05	0.5	1
dep	OR	1.00(0.94,1.06)	1.03(0.97,1.09)	1.02(0.96,1.08)	1.02(0.96,1.08)	1.03(0.97,1.09)	1.03(0.97,1.09)
	R2	2.07e-7	0.0002	0.0001	0.0001	0.0002	0.0002
	p	0.978	0.355	0.544	0.473	0.353	0.368
irb	OR	0.99(0.94,1.05)	1.02(0.96,1.08)	1.04(0.98,1.10)	1.05(1.00,1.11)	1.05(0.99,1.11)	1.04(0.99,1.10)
	R2	2.88e-5	0.0001	0.0005	0.0009	0.0007	0.0006
	p	0.743	0.518	0.189	0.0741	0.100	0.135
psy	OR	1.00(0.91,1.09)	1.04(0.95,1.13)	0.98(0.90,1.07)	1.05(0.96,1.14)	1.07(0.98,1.16)	1.06(0.97,1.16)
	R2	1.94e-6	0.0003	9.51e-5	0.0004	0.0009	0.0007
	p	0.944	0.369	0.624	0.302	0.135	0.173
apt	OR	0.99(0.94,1.05)	1.01(0.96,1.07)	1.03(0.97,1.09)	1.03(0,98,1.09)	1.02(0.97,1.08)	1.02(0.97,1.08)
	R2	2.70e-5	4.40e-5	0.0003	0.0004	0.0002	0.0001
	p	0.750	0.684	0.324	0.239	0.432	0.477
VAB	OR	1.01(0.95,1.07)	1.02(0.96,1.08)	1.04(0.98,1.10)	1.09(1.03,1.15)	1.08(1.02,1.15)	1.07(1.01.1.14)
	R2	3.97e-5	8.54e-5	0.0004	0.0022	0.0018	0.0016
	p	0.707	0.582	0.225	0.00526	0.0108	0.0179
POB	OR	1.01(0.95,1.07)	0.98(0.93,1.04)	1.01(0.96,1.07)	1.00(0.95,1.06)	0.99(0.94,1.05)	0.98(0.93,1.04)
	R2	3.06e-5	0.0001	4.73e-5	3.64e-6	3.31e-5	0.0001
	p	0.738	0.525	0.677	0.908	0.728	0.478
cog	OR	0.97(0.92,1.03)	0.99(0.93,1.04)	1.03(0.98,1.09)	1.02(0.97,1.08)	1.05(1.00,1.11)	1.05(1.00,1.11)
	R2	0.0003	6.09e-5	0.0004	0.0002	0.0010	0.0009
	p	0.324	0.632	0.237	0.414	0.0582	0.0691

dep = depression, irb = irritability, psy = psychosis, apt = apathy, VAB = violent/aggressive behaviour. POB= perseverative/obsessive behaviour. cog=cognitive impairment.

Supplementary Table S8: All PRS-symptom analyses for ASD PRS

Symptom	Statistic	PRS cutoff					
		0.0001	0.001	0.01	0.05	0.5	1
dep	OR	0.98(0.92,1.04)	0.98(0.93,1.04)	0.99(0.93,1.05)	0.99(0.93,1.05)	0.97(0.92,1.03)	0.97(0.92,1.03)
	R2	0.0002	8.62e-5	6.29e-5	3.78e-5	0.0003	0.0003
	p	0.446	0.574	0.631	0.710	0.304	0.291
irb	OR	1.00(0.94,1.05)	1.03(0.97,1.08)	1.01(0.95,1.07)	1.00(0.95,1.06)	1.00(0.95,1.06)	0.99(0.94,1.05)
	R2	6.72e-5	0.0002	1.72e-5	6.84e-8	8.63e-8	2.58e-5
	p	0.874	0.383	0.800	0.987	0.986	0.756
psy	OR	1.02(0.93,1.11)	1.04(0.95,1.13)	1.06(0.97,1.16)	1.04(0.95,1.13)	1.05(0.96,1.14)	1.06(0.97,1.16)
	R2	7.18e-5	0.0003	0.0008	0.0002	0.0004	0.0007
	p	0.670	0.396	0.168	0.434	0.300	0.184
apt	OR	1.03(0.97,1.09)	1.06(1.00,1.12)	1.03(0.97,1.08)	1.01(0.96,1.07)	1.00(0.94,1.05)	0.99(0.94,1.05)
	R2	0.0003	0.0010	0.0002	4.65e-5	8.93e-6	1.91e-5
	p	0.328	0.0499	0.345	0.676	0.855	0.789
VAB	OR	1.01(0.95,1.07)	1.06(1.00,1.12)	1.06(1,00,1.13)	1.03(0.98,1.10)	1.03(0.97,1.09)	1.03(0.97,1.09)
	R2	4.77e-5	0.0010	0.0011	0.0004	0.0002	0.0002
	p	0.681	0.0603	0.0451	0.255	0.379	0.366
POB	OR	0.96(0.91,1.02)	1.04(0.98,1.10)	1.02(0.97,1.08)	1.01(0.96,1.07)	1.01(0.96,1.07)	1.01(0.95,1.07)
	R2	0.0005	0.0004	0.0002	5.43e-5	5.67e-5	3.24e-5
	p	0.159	0.219	0.397	0.656	0.648	0.730
cog	OR	0.99(0.94,1.05)	1.05(1.00,1.11)	1.03(0.98,1.09)	1.03(0.98,1.09)	1.00(0.94,1.05)	1.00(0.94,1.05)
	R2	1.79e-5	0.0009	0.0003	0.0004	5.33e-6	1.40e-6
	p	0.795	0.0694	0.266	0.241	0.887	0.942

dep = depression, irb = irritability, psy = psychosis, apt = apathy, VAB = violent/aggressive behaviour. POB= perseverative/obsessive behaviour. cog=cognitive impairment.

Supplementary Table S9: All PRS-symptom analyses for OCD PRS

Symptom	Statistic	PRS cutoff					
		0.0001	0.001	0.01	0.05	0.5	1
dep	OR	1.04(0.98,1.10)	1.00(0.95,1.06)	1.09(1.03,1.15)	1.07(1.01,1.14)	1.08(1.01,1.14)	1.08(1.02,1.14)
	R2	0.0005	6.39e-6	0.0021	0.0016	0.0016	0.0017
	p	0.170	0.878	0.00516	0.0157	0.0142	0.0129
Irb	OR	1.02(0.96,1.08)	1.03(0.98,1.09)	1.02(0.96,1.08)	1.01(0.96,1.07)	1.03(0.97,1.09)	1.02(0.97,1.08)
	R2	9.30e-5	0.0004	0.0001	5.42e-5	0.0002	0.0002
	p	0.556	0.237	0.478	0.653	0.380	0.416
psy	OR	1.07(0.98,1.16)	0.98(0.90,1.07)	1.05(0.96,1.15)	0.99(0.91,1.08)	1.00(0.91,1.09)	1.00(0.91,1.09)
	R2	0.0009	7.44e-5	0.0005	2.50e-5	5.20e-6	1.09e-6
	p	0.137	0.665	0.284	0.802	0.909	0.958
apt	OR	1.01(0.96,1.07)	1.03(0.98,1.09)	0.99(0.94,1.05)	0.99(0.93,1.04)	1.00(0.95,1.06)	1.00(0.94,1.06)
	R2	7.20e-5	0.0004	1.13e-5	6.54e-5	1.60e-7	8.52e-7
	p	0.603	0.251	0.837	0.620	0.980	0.955
VAB	OR	1.02(0.96,1.08)	1.01(0.95,1.07)	1.03(0.97,1.10)	1.01(0.95,1.07)	1.01(0.95,1.07)	1.01(0.95,1.07)
	R2	0.0001	3.73e-5	0.0003	3.79e-5	2.50e-5	2.00e-5
	p	0.498	0.716	0.298	0.714	0.766	0.790
POB	OR	1.02(0.96,1.08)	1.07(1.01,1.13)	1.06(1.00,1.12)	1.05(0.99,1.11)	1.07(1.01,1.14)	1.07(1.01,1.13)
	R2	0.0001	0.0015	0.0009	0.0006	0.0015	0.0013
	p	0.515	0.0190	0.0667	0.126	0.0194	0.0281
cog	OR	1.00(0.95,1.06)	1.03(0.97,1.09)	1.01(0.96,1.07)	1.00(0.95,1.06)	1.00(0.95,1.06)	1.00(0.95,1.06)
	R2	4.78e-9	0.0003	4.94e-5	5.80e-6	7.45e-6	4.51e-6
	p	0.997	0.298	0.666	0.882	0.867	0.896

dep = depression, irb = irritability, psy = psychosis, apt = apathy, VAB = violent/aggressive behaviour. POB= perseverative/obsessive behaviour. cog=cognitive impairment.

Supplementary Table S10: All PRS-symptom analyses for AD PRS

Symptom	Statistic	PRS cutoff					
		0.0001	0.001	0.01	0.05	0.5	1
dep	OR	0.97(0.92,1.03)	0.98(0.92,1.03)	0.95(0.91,1.02)	0.98(0.93,1.04)	1.01(0.96,1.07)	1.02(0.96,1.08)
	R2	0.0003	0.0002	0.0005	8.41e-5	4.77e-5	0.0001
	p	0.303	0.427	0.171	0.579	0.676	0.490
irb	OR	1.00(0.95,1.06)	1.01(0.95,1.06)	0.99(0.93,1.04)	1.00(0.94,1.05)	1.03(0.97,1.09)	1.03(0.97,1.09)
	R2	7.63e-6	1.00e-5	7.27e-5	5.57e-6	0.0003	0.0002
	p	0.866	0.847	0.602	0.885	0.316	0.346
psy	OR	1.05(0.97,1.15)	1.10(1.01,1.20)	1.08(0.99,1.18)	1.06(0.97,1.16)	1.04(0.96,1.14)	1.04(0.96,1.14)
	R2	0.0006	0.0019	0.0013	0.0007	0.0003	0.0004
	p	0.234	0.0265	0.0729	0.181	0.350	0.326
apt	OR	1.00(0.94,1.05)	0.97(0.92,1.03)	0.99(0.93,1.04)	1.01(0.95,1.07)	1.03(0.98,1.09)	1.03(0.98,1.09)
	R2	5.56e-6	0.0003	6.26e-5	2.35e-5	0.0003	0.0003
	p	0.885	0.290	0.627	0.766	0.272	0.279
VAB	OR	1.00(0.94,1.06)	1.00(0.94,1.06)	1.01(0.95,1.07)	1.03(0.97,1.09)	1.02(0.97,1.09)	1.03(0.97,1.09)
	R2	3.17e-6	7.47e-6	1.56e-5	0.0003	0.0002	0.0002
	p	0.916	0.871	0.814	0.316	0.429	0.393
POB	OR	1.00(0.95,1.06)	1.04(0.98,1.10)	1.05(0.99,1.11)	1.05(0.99,1.11)	1.05(0.99,1.11)	1.04(0.99,1.11)
	R2	1.73e-6	0.0005	0.0007	0.0007	0.0008	0.0006
	p	0.937	0.165	0.114	0.119	0.0922	0.143
cog	OR	1.00(0.95,1.06)	1.02(0.96,1.08)	0.99(0.93,1.04)	1.00(0.94,1.05)	1.00(0.95,1.06)	1.01(0.95,1.07)
	R2	1.31e-7	0.0001	5.00e-5	8.62e-6	1.68e-6	2.89e-5
	p	0.982	0.503	0.664	0.857	0.937	0.741

dep = depression, irb = irritability, psy = psychosis, apt = apathy, VAB = violent/aggressive behaviour. POB= perseverative/obsessive behaviour. cog=cognitive impairment.

Supplementary Table S11: All PRS-symptom analyses for PD PRS

Symptom	Statistic	PRS cutoff					
		0.0001	0.001	0.01	0.05	0.5	1
dep	OR	0.99(0.94,1.05)	0.95(0.89,1.00)	0.96(0.91,1.02)	0.97(0.91,1.03)	1.00(0.94,1.06)	1.00(0.94,1.06)
	R2	1.60e-5	0.0009	0.0005	0.0003	4.90e-6	3.87e-6
	P	0.809	0.0620	0.169	0.287	0.893	0.905
irb	OR	1.02(0.97,1.08)	0.99(0.93,1.04)	0.99(0.94,1.05)	0.97(0.92,1.03)	0.98(0.93,1.04)	0.98(0.93,1.04)
	R2	0.0002	5.73e-5	2.24e-5	0.0002	0.0001	9.03e-5
	p	0.403	0.643	0.772	0.338	0.521	0.561
psy	OR	1.01(0.92,1.10)	0.94(0.87,1.03)	1.01(0.92,1.10)	1.01(0.93,1.10)	1.08(0.99,1.18)	1.08(0.99,1.18)
	R2	1.15e-5	0.0006	1.51e-5	1.78e-5	0.0011	0.0011
	p	0.865	0.203	0.845	0.832	0.0909	0.0960
apt	OR	1.01(0.96,1.07)	0.96(0.90,1.01)	0.99(0.93,1.04)	1.01(0.95,1.06)	0.99(0.93,1.04)	0.98(0.93,1.04)
	R2	2.83e-5	0.0007	6.36e-5	9.47e-6	5.47e-5	8.16e-5
	p	0.744	0.108	0.625	0.850	0.650	0.580
VAB	OR	1.05(0.99,1.12)	1.00(0.94,1.06)	0.99(0.93,1.05)	0.98(0.92,1.04)	0.98(0.93,1.04)	0.98(0.93,1.05)
	R2	0.0009	2.39e-6	5.05e-5	0.0002	8.71e-5	7.62e-5
	p	0.0798	0.927	0.672	0.419	0.578	0.603
POB	OR	1.03(0.98,1.09)	0.96(0.91,1.02)	1.00(0.95,1.06)	0.97(0.92,1.03)	0.98(0.93,1.04)	0.98(0.92,1.04)
	R2	0.0004	0.0006	7.50e-6	0.0002	0.0001	0.0002
	p	0.246	0.155	0.868	0.366	0.507	0.446
cog	OR	1.09(1.03,1.16)	1.02(0.96,1.08)	1.04(0.99,1.10)	1.01(0.96,1.07)	0.99(0.94,1.05)	0.99(0.93,1.04)
	R2	0.0026	0.0001	0.0006	5.40e-5	1.78e-5	5.59e-5
	p	0.00167	0.537	0.137	0.652	0.795	0.646

dep = depression, irb = irritability, psy = psychosis, apt = apathy, VAB = violent/aggressive behaviour. POB= perseverative/obsessive behaviour. cog=cognitive impairment.

Supplementary Table S12: All PRS-symptom analyses for intelligence PRS

Symptom	Statistic	PRS cutoff					
		0.0001	0.001	0.01	0.05	0.5	1
dep	OR	0.95(0.90,1.01)	0.93(0.87,0.98)	0.95(0.89,1.00)	0.94(0.89,1.00)	0.93(0.88,0.99)	0.93(0.88,0.99)
	R2	0.0008	0.0018	0.0010	0.0011	0.0016	0.0016
	P	0.0799	0.00951	0.0593	0.0410	0.0152	0.0166
irb	OR	0.92(0.87,0.97)	0.93(0.88,0.99)	0.91(0.86,0.96)	0.90(0.85,0.95)	0.90(0.85,0.95)	0.90(0.85,0.95)
	R2	0.0024	0.0016	0.0032	0.0034	0.0036	0.0037
	p	0.00306	0.0139	0.000532	0.000344	0.000246	0.000199
psy	OR	0.90(0.82,0.98)	0.93(0.85,1.01)	0.94(0.86,1.02)	0.95(0.87,1.04)	0.91(0.84,1.00)	0.91(0.84,0.99)
	R2	0.0024	0.0010	0.0008	0.0004	0.0017	0.0017
	p	0.0147	0.104	0.159	0.295	0.0405	0.0371
apt	OR	0.94(0.89,1.00)	0.94(0.88,0.99)	0.93(0.88,0.98)	0.91(0.86,0.96)	0.90(0.85,0.95)	0.90(0.85,0.95)
	R2	0.0012	0.0015	0.0020	0.0030	0.0039	0.0041
	p	0.0341	0.0166	0.00650	0.000852	0.000118	9.27e-5
VAB	OR	0.91(0.86,0.97)	0.90(0.85,0.96)	0.88(0.83,0.93)	0.89(0.84,0.95)	0.88(0.83,0.93)	0.88(0.83,0.93)
	R2	0.0027	0.0031	0.0050	0.0040	0.0048	0.0048
	p	0.00198	0.00101	2.44e-5	0.000156	4.09e-5	3.94e-5
POB	OR	0.96(0.91,1.02)	0.96(0.91,1.02)	0.93(0.88,0.98)	0.93(0.88,0.99)	0.94(0.89,1.00)	0.94(0.88,0.99)
	R2	0.0006	0.0006	0.0018	0.0016	0.0013	0.0014
	p	0.150	0.152	0.0103	0.0149	0.0318	0.0247
cog	OR	0.92(0.87,0.97)	0.91(0.86,0.96)	0.89(0.84,0.94)	0.90(0.85,0.95)	0.90(0.85,0.96)	0.90(0.85,0.96)
	R2	0.0025	0.0031	0.0045	0.0040	0.0034	0.0033
	p	0.00213	0.000633	3.90e-5	0.000108	0.000378	0.000384

dep = depression, irb = irritability, psy = psychosis, apt = apathy, VAB = violent/aggressive behaviour. POB= perseverative/obsessive behaviour. cog=cognitive impairment.

Supplementary Table S13: HD symptom – polygenic risk score associations correcting for significant clinical covariates

SYMPTOM	Risk score	PRS CUT-OFF	OR	R^2 (%)	AUC	P VALUE
Depression	MDD	0.01	1.17	0.73	0.600	2.17×10^{-7}
	Schizophrenia	0.05	1.12	0.37	0.595	2.30×10^{-4}
	BPD	0.01	1.11	0.31	0.593	6.84×10^{-4}
	Intelligence	0.001	0.94	0.13	0.591	0.0286
Irritability	Schizophrenia	0.05	1.16	0.70	0.583	4.64×10^{-7}
	MDD	0.001	1.12	0.39	0.579	1.68×10^{-4}
	Intelligence	1	0.91	0.30	0.577	9.67×10^{-4}
Psychosis	Schizophrenia	0.001	1.18	0.55	0.604	2.40×10^{-4}
	MDD	0.05	1.13	0.30	0.602	6.80×10^{-3}
	Alzheimer's	0.001	1.11	0.24	0.598	0.0164
	Intelligence	0.0001	0.91	0.19	0.600	0.0297
VAB	Intelligence	0.01	0.88	0.45	0.608	7.17×10^{-5}
	Schizophrenia	0.05	1.14	0.48	0.608	4.22×10^{-5}
	MDD	0.001	1.11	0.33	0.607	6.74×10^{-4}
	BPD	0.001	1.11	0.32	0.605	8.61×10^{-4}
	ADHD	0.05	1.09	0.22	0.604	5.70×10^{-3}
	ASD	0.01	1.06	0.08	0.603	0.0851
POB	Schizophrenia	0.01	1.11	0.36	0.583	3.13×10^{-4}
	BPD	0.5	1.07	0.14	0.582	0.0262
	Intelligence	0.01	0.93	0.18	0.582	0.0100
	MDD	0.5	1.07	0.12	0.580	0.0329
	OCD	0.001	1.08	0.18	0.580	0.0111
Apathy	Intelligence	1	0.90	0.38	0.576	1.99×10^{-4}
	MDD	0.05	1.08	0.18	0.575	0.0103
	BPD	0.001	1.07	0.14	0.574	0.0245
	Schizophrenia	0.0001	1.07	0.16	0.575	0.0145
	ASD	0.001	1.05	0.07	0.575	0.0977
Cognitive impairment	Intelligence	0.01	0.89	0.41	0.636	7.39×10^{-5}
	Parkinson's	0.0001	1.10	0.25	0.633	2.19×10^{-3}
	MDD	0.5	1.09	0.24	0.634	2.41×10^{-3}

VAB= violent/aggressive behaviour, POB=perserverative/obsessive behaviour. MDD= major depressive disorder. BPD= bipolar disorder. ASD= autism spectrum disorder. ADHD = attention deficit hyperactivity disorder. Analyses include the covariates significantly associated with each symptom in Table 2. R^2= Nagelkerke R^2 attributable solely to the PRS . OR= Odds ratio for presence of symptom associated with 1 s.d. increase in PRS. AUC refers to the combined prediction of covariates and PRS.

Supplementary Table S14: Testing for sex differences in PRS odds ratios for symptoms with both a significant sex difference and a significant PRS association

Symptom	PRS	cutoff	All		Males		Females		M vs F P value
			OR	p	OR	p	OR	P	
depression	SCZ	0.05	1.12	2.47e-4	1.16	3.79e-4	1.08	0.0894	0.207
	BPD	0.01	1.11	3.06e-4	1.17	1.96e-4	1.06	0.180	0.114
	MDD	0.01	1.17	9.01e-8	1.18	4.14e-5	1.13	3.51e-3	0.386
	INT	0.001	0.93	9.51e-3	0.92	0.0342	0.94	0.135	0.719
irritability	SCZ	0.05	1.17	1.03e-7	1.15	1.44e-3	1.18	2.74e-5	0.598
	MDD	0.001	1.12	4.42e-5	1.13	4.19e-3	1.12	6.18e-3	0.869
	INT	1	0.90	1.99e-4	0.93	0.0966	0.87	3.42e-4	0.202
VAB	SCZ	0.05	1.13	5.46e-5	1.14	3.49e-3	1.13	6.59e-3	0.904
	BPD	0.001	1.11	5.35e-4	1.07	0.0961	1.16	7.73e-4	0.203
	ADHD	0.05	1.09	5.26e-3	1.08	0.0711	1.12	0.0131	0.588
	MDD	0.001	1.13	8.45e-5	1.10	0.0243	1.15	1.41e-3	0.436
	INT	0.01	0.88	2.44e-5	0.88	2.22e-3	0.88	4.08e-3	0.999

VAB = violent/aggressive behaviour.

Supplementary Table S15: Association of PRS with number of symptoms

PRS	Number of symptoms 0 (473)	1 (700)	2 (858)	3 (952)	4 (865)	5 (715)	6 (433)	7 (164)	P(fac)	P(quant)
SCZ-0001	-0.090	-0.076	-0.024	0.002	0.051	-0.010	0.142	0.118	0.00285	4.80e-5
SCZ-001	-0.091	-0.076	-0.027	-0.002	0.039	0.024	0.162	0.109	0.00134	6.48e-6
SCZ-01	-0.114	-0.094	-0.064	0.024	0.018	0.083	0.167	0.138	5.62e-6	5.06e-9
SCZ-05	-0.128	-0.076	-0.068	0.023	0.026	0.069	0.164	0.166	5.88e-6	4.45e-9
SCZ-5	-0.108	-0.068	-0.064	0.036	0.003	0.070	0.132	0.206	4.62e-5	1.11e-7
SCZ-1	-0.112	-0.069	-0.059	0.032	0.005	0.069	0.128	0.024	6.67e-5	1.25e-7
BIP-0001	-0.039	-0.069	0.001	-0.004	0.047	-0.014	0.065	-0.021	0.327	0.067
BIP-001	-0.071	-0.066	-0.024	0.024	0.042	0.005	0.090	0.026	0.109	0.00345
BIP-01	-0.091	-0.053	-0.020	0.039	0.012	0.024	0.092	0.032	0.102	0.00268
BIP-05	-0.086	-0.035	-0.002	0.028	-0.011	0.016	0.069	0.101	0.250	0.0118
BIP-5	-0.067	-0.030	0.008	-0.005	-0.032	0.034	0.047	0.107	0.396	0.0348
BIP-1	-0.061	-0.029	0.0002	-0.011	-0.033	0.040	0.044	0.120	0.354	0.0273
MDD-0001	-0.127	-0.082	-0.003	0.032	-0.010	0.067	-0.006	0.102	0.0122	0.000897
MDD-001	-0.178	-0.069	-0.014	0.052	-0.008	0.081	0.031	0.192	4.32e-5	2.583e-6
MDD-01	-0.167	-0.074	-0.015	-0.004	0.034	0.059	0.053	0.261	2.46e-5	1.34e-7
MDD-05	-0.175	-0.082	-0.023	-0.014	0.013	0.067	0.084	0.206	3.25e-5	4.58e-8
MDD-5	-0.133	-0.117	-0.011	-0.013	0.023	0.058	0.077	0.263	1.34e-5	4.17e-8
MDD-1	-0.136	-0.116	-0.011	-0.011	0.024	0.057	0.072	0.253	2.15e-5	6.35e-8
ADHD-0001	-0.030	0.016	0.012	-0.002	0.022	-0.034	-0.022	0.020	0.945	0.800
ADHD-001	-0.024	0.000	0.005	-0.024	0.034	0.004	-0.033	0.076	0.867	0.602
ADHD-01	-0.059	-0.039	0.006	0.008	0.030	0.017	0.015	-0.046	0.762	0.203
ADHD-05	-0.061	-0.043	0.008	-0.020	0.039	0.002	0.038	0.049	0.580	0.0561
ADHD-5	-0.056	-0.032	-0.014	-0.034	0.079	0.002	0.040	0.020	0.235	0.0464
ADHD-1	-0.055	-0.029	-0.009	-0.035	0.083	-0.002	0.028	0.009	0.238	0.0821
ASD-0001	-0.026	0.089	-0.040	-0.010	-0.014	-0.001	-0.069	0.123	0.104	0.586
ASD-001	-0.004	0.040	-0.041	-0.043	-0.021	0.079	0.052	0.120	0.101	0.102
ASD-01	-0.015	0.024	-0.045	-0.022	-0.013	0.055	0.048	-0.025	0.547	0.301
ASD-05	-0.002	0.015	-0.024	-0.074	-0.004	0.045	0.025	0.004	0.426	0.470
ASD-5	0.041	0.010	-0.034	-0.076	-0.013	0.010	0.039	-0.026	0.397	0.988
ASD-1	0.044	0.008	-0.034	-0.075	-0.017	0.008	0.038	-0.025	0.409	0.958
OCD-0001	-0.066	0.020	-0.006	0.017	0.010	-0.011	0.009	0.152	0.490	0.209
OCD-001	-0.046	-0.001	0.001	0.005	0.020	-0.007	0.053	0.073	0.856	0.169
OCD-01	-0.031	-0.041	0.023	-0.010	0.050	0.009	0.071	0.005	0.523	0.0895
OCD-05	-0.035	-0.017	0.025	0.007	0.023	0.017	0.053	-0.092	0.740	0.453
OCD-5	-0.038	-0.021	0.021	0.011	-0.001	0.026	0.130	-0.101	0.159	0.137
OCD-1	-0.033	-0.025	0.022	0.006	-0.002	0.028	0.126	-0.107	0.169	0.157
INT-0001	0.114	0.101	-0.033	0.057	-0.038	-0.025	-0.038	-0.230	2.17e-4	4.15e-5
INT-001	0.100	0.112	-0.020	0.025	-0.025	-0.030	-0.086	-0.161	1.88e-3	2.81e-5
INT-01	0.121	0.118	-0.006	0.030	-0.038	-0.054	-0.113	-0.120	1.61e-4	6.25e-7
INT-05	0.123	0.116	-0.003	0.078	-0.074	-0.031	-0.136	-0.072	7.19e-6	5.26e-7
INT-5	0.093	0.113	0.014	0.059	-0.090	-0.027	-0.156	-0.103	1.24e-6	7.46e-8
INT-1	0.092	0.137	0.016	0.062	-0.089	-0.029	-0.160	-0.097	7.93e-7	5.78e-8

Number of symptoms analysed both as an 8-level factor, denoted by p(fac) and as a quantitative variable, denoted by p(quant). Numbers in columns headed by symptom count are the mean PRS among individuals with that number of symptoms.

Supplementary Table S16: Association of SCZ PRS with symptoms after correction for the number of other symptoms present (general severity)

PRS	psy	dep	irb	VAB	apt	POB	cog
SCZ-0001	**0.00416**	0.234	0.138	0.914	0.458	0.187	0.253
SCZ-001	**0.00370**	0.158	0.0662	0.717	0.850	**0.0207**	0.470
SCZ-01	**0.0157**	0.104	**0.00293**	0.227	0.548	0.0589	0.929
SCZ-05	**0.0383**	0.0426	**0.000904**	0.0644	0.339	0.269	0.975
SCZ-5	**0.0249**	0.0844	**0.00370**	0.189	0.204	**0.0432**	0.694
SCZ-1	**0.0295**	0.0824	**0.00419**	0.176	0.195	**0.0412**	0.733

Nominally significant associations shown in **bold**.

dep = depression, irb = irritability, psy = psychosis, apt = apathy, VAB = violent/aggressive behaviour. POB= perseverative/obsessive behaviour. cog=cognitive impairment.

Supplementary Table S17: Association of MDD PRS with symptoms after correction for the number of other symptoms present (general severity)

PRS	psy	dep	irb	VAB	apt	POB	cog
MDD-0001	0.350	**0.00369**	**0.00298**	0.0667	0.308	0.193	0.600
MDD-001	0.113	**0.00306**	**0.0183**	**0.0234**	0.468	0.243	0.318
MDD-01	0.152	**$2.41x10^{-5}$**	0.0589	0.0588	0.885	0.602	0.945
MDD-05	0.115	**$3.46x10^{-4}$**	0.196	0.258	0.759	0.749	0.202
MDD-5	0.234	**$9.34x10^{-4}$**	0.388	0.101	0.909	0.783	0.195
MDD-1	0.290	**$5.44x10^{-4}$**	0.336	0.0906	0.863	0.898	0.246

Nominally significant associations shown in **bold**.

dep = depression, irb = irritability, psy = psychosis, apt = apathy, VAB = violent/aggressive behaviour. POB= perseverative/obsessive behaviour. cog=cognitive impairment.

Supplementary Table S18: Association of intelligence PRS with symptoms after correction for the number of other symptoms present (general severity)

PRS	psy	dep	irb	VAB	apt	POB	cog
INT-0001	0.179	0.862	0.277	0.110	0.544	0.842	**0.036**
INT-001	0.637	0.221	0.701	0.088	0.429	0.729	**0.022**
INT-01	0.988	0.848	0.167	**0.019**	0.570	0.551	**0.0078**
INT-05	0.696	0.728	0.149	0.069	0.149	0.654	**0.0113**
INT-5	0.553	0.448	0.166	**0.044**	0.069	0.939	**0.0444**
INT-1	0.535	0.487	0.149	**0.047**	0.065	0.981	**0.0476**

Nominally significant associations shown in **bold**.

dep = depression, irb = irritability, psy = psychosis, apt = apathy, VAB = violent/aggressive behaviour. POB= perseverative/obsessive behaviour. cog=cognitive impairment.

Guidelines for the age at onset questionnaire for EHDN-HD Registry patients

This questionnaire will capture essential and detailed information on when signs and symptoms that may be related or unrelated to HD first appear. Use the suggested wording below as a guide, but ask any other questions you need to try and date the onset as accurately as possible. Ask the patient first, then the carer (explain to the carer at the beginning that you will give them a chance to state their views after you have first heard from the patient) and finally record your own judgement about onset, taking account of what has been said by both patient and carer, and also what you can find in the case notes (or any other independent sources of information). It is not uncommon to find a record by a genetic counsellor who saw the patient and noticed choreiform movements, or an interview with a relative who mentioned that the family think the patient is affected, and this information may pre-date the estimates of either the patient or the carer who is acting as your informant on this occasion. Use your own judgement about the evidence, but avoid making guesses based only on your own experience of how long the average patient would need to be affected to reach this stage in the illness.

1. Motor symptoms:
   When did you first notice any motor symptoms of HD, such as fidgety movements or clumsiness? Can you remember a particular occasion when this happened and you first realised you might be developing the family illness? What were you doing at the time? Where were you? Did you mention it to anyone else? (Try to identify any landmark events in the patient's life around that time, such as holidays, births, marriages & deaths in the family, etc, which might help to anchor the onset date by asking whether the onset was before or after that particular landmark)

2. Depression:
   Have you ever suffered from depression for a period of 4 weeks or more? Was there ever a time you felt so low that you thought about ending it all? Have you been treated with antidepressants? When was the first time this happened to you (doesn't necessarily have to have been medicated in the first or any subsequent instance)?

3. Irritability and aggression:
   Have there been times when you were irritable, bad tempered or 'cranky' for a period of 4 weeks or more? Have you had difficulty keeping control of your temper? When did this begin? If so, how bad was the worst ever episode? Has there been overt aggression? If so when did this first occur?

4. Apathy:
   Have you noticed any change in your level of motivation? For example, has there ever been a period of four weeks or more when you lost interest in things that usually matter to you, or could not be bothered with activities you usually enjoy, or needed pushing to get around to jobs that need to be done, or spent a lot of time sitting around doing nothing? Did this ever happen at a time when you were not also feeling depressed? When did you first notice this change?

5. Perseverative or Obsessive/Compulsive behaviours:
   Have you ever been troubled by recurrent thoughts, fears or mental images that came back over and over again, or had urges to do something over and over (e.g. double-checking, counting things, or washing your hands repeatedly)? Or perhaps found yourself unable to stop thinking about something, so that friends said you were getting it out of proportion, or getting

a ‘bee in your bonnet’? If so, how bad was the worst ever episode? When was the first time this happened to you?

6. Presence of psychosis:
   Has the patient ever reported symptoms resembling schizophrenia e.g. paranoid thinking, delusions and hearing voices. If so when was the first episode?

7. Cognitive symptoms:
   Have you ever noticed any problems with your memory, or your ability to concentrate on things? Have you found it more difficult than it used to be to organise things, or perhaps noticed that it takes longer to get things done nowadays? Has anybody commented on this, or maybe criticised your performance at work or when doing chores at home? Can you remember the first time this happened? (probe as above and look for evidence such as job changes etc. which might suggest early cognitive difficulties)

European Huntington's Disease Network REGISTRY Study Investigators

Registry Steering committee: Anne-Catherine Bachoud-Lévi, Anna Rita Bentivoglio, Ida Biunno, Raphael M Bonelli, Jean-Marc Burgunder, Stephen B Dunnett, Joaquim J Ferreira, Olivia J. Handley, Arvid Heiberg, Torsten Illmann, G Bernhard Landwehrmeyer, Jamie Levey, Maria A. Ramos-Arroyo, Jørgen E Nielsen, Susana Pro Koivisto, Markku Päivärinta, Raymund A.C. Roos, Ana Rojo Sebastián, Sarah J Tabrizi, Wim Vandenberghe, Christine Verellen-Dumoulin, Tereza Uhrova, Jan Wahlström, Jacek Zaremba

Language coordinators: Verena Baake, Katrin Barth, Monica Bascuñana Garde, Sabrina Betz, Reineke Bos, Jenny Callaghan, Adrien Come, Leonor Correia Guedes, Daniel Ecker, Ana Maria Finisterra, Ruth Fullam, Mette Gilling, Lena Gustafsson, Olivia J. Handley, Carina Hvalstedt, Christine Held, Kerstin Koppers, Claudia Lamanna, Matilde Laurà, Asunción Martínez Descals, Saül Martinez-Horta, Tiago Mestre, Sara Minster, Daniela Monza, Lisanne Mütze, Martin Oehmen, Michael Orth, Hélène Padieu, Laurent Paterski, Nadia Peppa, Susana Pro Koivisto, Martina Di Renzo, Amandine Rialland, Niini Røren, Pavla Šašinková, Erika Timewell, Jenny Townhill, Patricia Trigo Cubillo, Wildson Vieira da Silva, Marleen R van Walsem, Carina Whalstedt, Marie-Noelle Witjes-Ané , Grzegorz Witkowski, Abigail Wright, Daniel Zielonka, Eugeniusz Zielonka, Paola Zinzi

AUSTRIA

Graz (Medizinische Universitäts Graz, Psychiatrie): Raphael M Bonelli, Sabine Lilek, Karen Hecht, Brigitte Herranhof, Anna Holl (formerly Hödl), Hans-Peter Kapfhammer, Michael Koppitz, Markus Magnet, Nicole Müller, Daniela Otti, Annamaria Painold, Karin Reisinger, Monika Scheibl, Helmut Schöggl, Jasmin Ullah

Innsbruck (Universitätsklinik Innsbruck, Neurologie): Eva-Maria Braunwarth, Florian Brugger, Lisa Buratti, Eva-Maria Hametner, Caroline Hepperger, Christiane Holas, Anna Hotter, Anna Hussl, Christoph Müller, Werner Poewe, Klaus Seppi, Fabienne Sprenger, Gregor Wenning

BELGIUM

Bierbeek: Andrea Boogaerts, Godelinde Calmeyn, Isabelle Delvaux, Dirk Liessens, Nele Somers

Charleroi (Institut de Pathologie et de Génétique (IPG)): Michel Dupuit, Cécile Minet, Dominique van Paemel, Pascale Ribaï, Christine Verellen-Dumoulin

Leuven: (Universitair Ziekenhuis Gasthuisberg,): Andrea Boogaerts, Wim Vandenberghe, Dimphna van Reijen

CZECH REPUBLIC

Prague (Extrapyramidové centrum, Neurologická klinika, 1. LF UK a VFN): Jiří Klempíř, Veronika Majerová, Jan Roth, Irena Stárková

DENMARK

Copenhagen (Neurogenetics Clinic, Danish Dementia Research Centre, Rigshospitalet, University of Copenhagen): Lena E. Hjermind, Oda Jacobsen, Jørgen E Nielsen, Ida Unmack Larsen, Tua Vinther-Jensen

FINLAND

Turku-Suvituuli (Rehabilitation Centre Suvituuli): Heli Hiivola, Hannele Hyppönen, Kirsti Martikainen, Katri Tuuha

FRANCE

Angers (Centre de référence des maladies neurogénétique- CHU d'Angers): Philippe Allain, Dominique Bonneau, Marie Bost, Bénédicte Gohier, Marie-Anne Guérid, Audrey Olivier, Adriana Prundean, Clarisse Scherer-Gagou, Christophe Verny

Bordeaux (Hôpital CHU Pellegrin): Blandine Babiloni, Sabrina Debruxelles, Charlotte Duché, Cyril Goizet, Laetitia Jameau, Danielle Lafoucrière, Umberto Spampinato

Lille-Amiens:

Lille (CHRU Roger Salengro) : Rekha Barthélémy, Christelle De Bruycker, Maryline Cabaret, Anne-Sophie Carette, Eric Decorte Luc Defebvre, Marie Delliaux, Arnaud Delval, Alain Destee, Kathy Dujardin, Marie-Hélène Lemaire, Sylvie Manouvrier, Mireille Peter, Lucie Plomhouse, Bernard Sablonnière, Clémence Simonin, Stéphanie Thibault-Tanchou, Isabelle Vuillaume

Amiens (CHU Nord) : Marcellin Bellonet, Hassan Berrissoul, Stéphanie Blin, Françoise Courtin, Cécile Duru, Véronique Fasquel, Olivier Godefroy, Pierre Krystkowiak, Béatrice Mantaux, Martine Roussel, Sandrine Wannepain

Marseille (Hôpital La Timone) : Jean-Philippe Azulay, Marie Delfini, Alexandre Eusebio, Frédérique Fluchere, Laura Mundler

Strasbourg (Hôpital Civil) : Mathieu Anheim, Celine Julié, Ouhaid Lagha Boukbiza, Nadine Longato, Gabrielle Rudolf, Christine Tranchant, Marie-Agathe Zimmermann

GERMANY

Aachen (Universitätsklinikum Aachen, Neurologische Klinik): Christoph Michael Kosinski, Eva Milkereit, Daniela Probst, Kathrin Reetz, Christian Sass, Johannes Schiefer, Christiane Schlangen, Cornelius J. Werner

Berlin (Klinik und Poliklinik für Neurologie - Charité - Universitätsmedizin Berlin): Harald Gelderblom, Josef Priller, Harald Prüß, Eike Jakob Spruth

Bochum (Huntington-Zentrum (NRW) Bochum im St. Josef-Hospital): Gisa Ellrichmann, Lennard Herrmann, Rainer Hoffmann, Barbara Kaminski, Peter Kotz, Christian Prehn, Carsten Saft

Dinslaken (Reha Zentrum in Dinslaken im Gesundheitszentrums Lang): Herwig Lange, Robert Maiwald

Dresden (Universitätsklinikum Carl Gustav Carus an der Technischen Universität Dresden, Klinik und Poliklinik für Neurologie): Matthias Löhle, Antonia Maass, Simone Schmidt, Cecile Bosredon, Alexander Storch, Annett Wolz, Martin Wolz

Freiburg (Universitätsklinik Freiburg, Neurologie): Philipp Capetian, Johann Lambeck, Birgit Zucker

Hamburg (Universitätsklinikum Hamburg-Eppendorf, Klinik und Poliklinik für Neurologie): Kai Boelmans, Christos Ganos, Walburgis Heinicke, Ute Hidding, Jan Lewerenz, Alexander Münchau, Michael Orth, Jenny Schmalfeld, Lars Stubbe, Simone Zittel

Hannover (Neurologische Klinik mit Klinischer Neurophysiologie, Medizinische Hochschule Hannover): Gabriele Diercks, Dirk Dressler, Heike Gorzolla, Christoph Schrader, Pawel Tacik

Itzehoe (Schwerpunktpraxis Huntington, Neurologie und Psychiatrie): Michael Ribbat

Marburg KPP (Klinik für Psychiatrie und Psychotherapie Marburg-Süd): Bernhard Longinus

Marburg Uni (Universität Marburg, Neurologie): Katrin Bürk, Jens Carsten Möller, Ida Rissling

München (Huntington-Ambulanz im Neuro-Kopfzentrum - Klinikum rechts der Isar der

Neurologischen Klinik und Poliklinik der Technischen Universität München): Mark Mühlau, Alexander Peinemann, Michael Städtler, Adolf Weindl, Juliane Winkelmann, Cornelia Ziegler

Münster (Universitätsklinikum Münster, Klinik und Poliklinik für Neurologie): Natalie Bechtel, Heike Beckmann, Stefan Bohlen, Eva Hölzner, Herwig Lange, Ralf Reilmann, Stefanie Rohm, Silke Rumpf , Sigrun Schepers, Natalia Weber

Taufkirchen (Isar-Amper-Klinikum - Klinik Taufkirchen (Vils)): Matthias Dose, Gabriele Leythäuser, Ralf Marquard, Tina Raab, Alexandra Wiedemann

Ulm (Universitätsklinikum Ulm, Neurologie): Katrin Barth, Andrea Buck, Julia Connemann, Daniel Ecker, Carolin Geitner, Christine Held, Andrea Kesse, Bernhard Landwehrmeyer, Christina Lang, Jan Lewerenz, Franziska Lezius, Solveig Nepper, Anke Niess, Michael Orth, Ariane Schneider, Daniela Schwenk, Sigurd Süßmuth, Sonja Trautmann, Patrick Weydt

ITALY

Bari Clinica Neurologica - Neurophysiopatology of Pain Unit UNIVERSITA' DI BARI): Claudia Cormio, Vittorio Sciruicchio, Claudia Serpino, Marina de Tommaso

Bologna (DIBINEM - Alma Mater Studiorum - Università di Bologna; IRCCS Istituto delle Scienze Neurologiche di Bologna): Sabina Capellari, Pietro Cortelli, Roberto Galassi, Rizzo Giovanni, Roberto Poda, Cesa Scaglione

Florence (Dipartimento di Scienze Neurologiche e Psichiatriche Universita' degli Studi di Firenze-Azienda Ospedaliera Universitaria Careggi): Elisabetta Bertini, Elena Ghelli, Andrea Ginestroni, Francesca Massaro, Claudia Mechi, Marco Paganini, Silvia Piacentini, Silvia Pradella, Anna Maria Romoli, Sandro Sorbi

Genoa (Dipartimento di Neuroscienze, Riabilitazione, Oftalmologia, Genetica e Scienze Materno-Infantili, Università di Genova): Giovanni Abbruzzese, Monica Bandettini di Poggio, Giovanna Ferrandes, Paola Mandich, Roberta Marchese

Milan (Fondazione IRCCS Istituto Neurologico Carlo Besta):
Alberto Albanese, Daniela Di Bella, Anna Castaldo, Stefano Di Donato, Cinzia Gellera, Silvia Genitrini, Caterina Mariotti, Daniela Monza, Lorenzo Nanetti, Dominga Paridi, Paola Soliveri, Chiara Tomasello

Naples (Dipartimento di Neuroscienze, Scienze Riproduttive e Odontostomatologiche, Università Federico II): Giuseppe De Michele, Luigi Di Maio, Marco Massarelli, Silvio Peluso, Alessandro Roca, Cinzia Valeria Russo, Elena Salvatore, Pierpaolo Sorrentino

Pozzilli (IS) (Centro di Neurogenetica e Malattie Rare - IRCCS Neuromed): Enrico Amico, Mariagrazia Favellato, Annamaria Griguoli, Irene Mazzante, Martina Petrollini, Ferdinando Squitieri and **Rome (Lega Italiana Ricerca Huntington e malattie correlate - onlus / www.LIRH.it):** Barbara D'Alessio, Chiara Esposito

Rome (Istituto di Farmacologia Traslazionale & Istituto di Scienze e Tecnologie della Cognizione /CNR, Istituto di Neurologia Università Cattolica del Sacro Cuore): Anna Rita Bentivoglio, Marina Frontali, Arianna Guidubaldi, Tamara Ialongo, Gioia Jacopini, Carla Piano, Silvia Romano, Francesco Soleti, Maria Spadaro, Paola Zinzi

NETHERLANDS

Enschede (Medisch Spectrum Twente): Monique S.E. van Hout, Marloes E. Verhoeven, Jeroen P.P. van Vugt, A. Marit de Weert

Groningen (Polikliniek Neurologie): J.J.W. Bolwijn, M. Dekker, B. Kremer, K.L. Leenders, J.C.H. van Oostrom

Leiden (Leiden University Medical Centre (LUMC)): Simon J. A. van den Bogaard, Reineke Bos, Eve M. Dumas, Ellen P. ‘t Hart, Raymund A.C. Roos

Nijmegen (Universitair Medisch Centrum St. Radboud, Neurology): Berry Kremer, C.C.P. Verstappen

NORWAY

Oslo University Hospital (Rikshospitalet, Dept. of Medical Genetics and Dept. of Neurology): Olaf Aaserud, Jan Frich C., Arvid Heiberg, Marleen R. van Walsem, Ragnhild Wehus

Oslo University Hospital (Ulleval, Dept. of Medical Genetics and Dept.of Neurorehabilitation): Kathrine Bjørgo, Madeleine Fannemel, Per F. Gørvell, Eirin Lorentzen, Susana Pro Koivisto, Lars Retterstøl, Bodil Stokke

Trondheim (St. Olavs Hospital): Inga Bjørnevoll, Sigrid Botne Sando

POLAND

Gdansk (St. Adalbert Hospital, Gdansk, Medical University of Gdansk, Neurological and Psychiatric Nursing Dpt.): Artur Dziadkiewicz, Malgorzata Nowak, Piotr Robowski, Emilia Sitek, Jaroslaw Slawek, Witold Soltan, Michal Szinwelski

Katowice (Medical University of Silesia, Katowice): Magdalena Blaszcyk, Magdalena Boczarska-Jedynak, Ewelina Ciach-Wysocka, Agnieszka Gorzkowska, Barbara Jasinska-Myga, Gabriela Kłodowska–Duda, Gregorz Opala, Daniel Stompel

Krakow (Krakowska Akademia Neurologii): Krzysztof Banaszkiewicz, Dorota Boćwińska, Kamila Bojakowska-Jaremek, Małgorzata Dec, Malgorzata Krawczyk, Monika Rudzińska, Elżbieta Szczygieł, Andrzej Szczudlik, Anna Wasielewska, Magdalena Wójcik

Poznan (Poznan University of Medical Sciences, Poland): Anna Bryl, Anna Ciesielska, Aneta Klimberg, Jerzy Marcinkowski, Husam Samara, Justyna Sempołowicz, Daniel Zielonka

Warsaw-MU (Medical University of Warsaw, Neurology): Anna Gogol (formerly Kalbarczyk), Piotr Janik, Hubert Kwiecinski, Zygmunt Jamrozik

Warsaw-IPiN (Institute of Psychiatry and Neurology Dep. of Genetics, First Dep. of Neurology): Jakub Antczak, Katarzyna Jachinska, Wioletta Krysa, Maryla Rakowicz, Przemyslaw Richter, Rafal Rola, Danuta Ryglewicz, Halina Sienkiewicz-Jarosz, Iwona Stępniak, Anna Sułek, Grzegorz Witkowski, Jacek Zaremba, Elzbieta Zdzienicka, Karolina Zieora-Jakutowicz

PORTUGAL

Coimbra (Hospital Universitário de Coimbra): Cristina Januário, Filipa Júlio

Lisbon (Clinical Pharmacology Unit, Instituto de Medicina Molecular, Faculty of Medicine, University of Lisbon): Joaquim J Ferreira, Miguel Coelho, Leonor Correia Guedes, Tiago Mendes, Tiago Mestre, Anabela Valadas

Porto (Hospital de São João, (Faculdade de Medicina da Universidade do Porto)): Carlos Andrade, Miguel Gago, Carolina Garrett, Maria Rosália Guerra.

SPAIN

Badajoz (Hospital Infanta Cristina): Carmen Durán Herrera, Patrocinio Moreno Garcia

Barcelona-Hospital Mútua de Terrassa: Miquel Aguilar Barbera, Dolors Badenes Guia, Laura Casas Hernanz , Judit López Catena, Pilar Quiléz Ferrer, Ana Rojo Sebastián, Gemma Tome Carruesco

Barcelona-Bellvitge (Hospital Universitari de Bellvitge): Jordi Bas, Núria Busquets, Matilde Calopa

Barcelona-Merced (Hospital Mare de Deu de La Merced): Misericordia Floriach Robert, Celia Mareca Viladrich, Jesús Miguel Ruiz Idiago, Antonio Villa Riballo

Burgos (Servicio de Neurología Hospital General Yagüe): Esther Cubo, Cecilia Gil Polo, Natividad Mariscal Perez, Jessica Rivadeneyra

Granada (Hospital Universitario San Cecilio, Neurología): Francisco Barrero, Blas Morales

Madrid-Clinico (Hospital Clínico Universitario San Carlos): María Fenollar, Rocío García-Ramos García, Paloma Ortega, Clara Villanueva

Madrid RYC (Hospital Ramón y Cajal, Neurología): Javier Alegre, Mónica Bascuñana, Juan Garcia Caldentey, Marta Fatás Ventura, Guillermo García Ribas, Justo García de Yébenes, José Luis López-Sendón Moreno, Patricia Trigo Cubillo

Madrid FJD (Madrid-Fundación Jiménez Díaz): Javier Alegre, Fernando Alonso Frech, Justo García de Yébenes, Pedro J García Ruíz, Asunción Martínez-Descals, Rosa Guerrero, María José Saiz Artiga, Vicenta Sánchez

Murcia (Hospital Universitario Virgen de la Arrixaca): María Fuensanta Noguera Perea, Lorenza Fortuna, Salvadora Manzanares, Gema Reinante, María Martirio Antequera Torres, Laura Vivancos Moreau

Oviedo (Hospital Central de Asturias): Sonia González González, Luis Menéndez Guisasola, Carlos Salvador, Esther Suaréz San Martín

Palma de Mallorca (Hospital Universitario Son Espases): Inés Legarda Ramirez, Aranzazú Gorospe, Mónica Rodriguez Lopera, Penelope Navas Arques, María José Torres Rodríguez, Barbara Vives Pastor

Pamplona (Complejo Hospitalario de Navarra): Itziar Gaston, Maria Dolores Martinez-Jaurrieta, Maria A. Ramos-Arroyo

Sevilla ("Hospital Virgen Macarena"): Jose Manuel Garcia Moreno, Carolina Mendez Lucena, Fatima Damas Hermoso, Eva Pacheco Cortegana, José Chacón Peña, Luis Redondo

Sevilla (Hospital Universitario Virgen del Rocío): Fátima Carrillo, María Teresa Cáceres, Pablo Mir, María José Lama Suarez, Laura Vargas-González

Valencia (Hospital la Fe): Maria E. Bosca, Francisco Castera Brugada, Juan Andres Burguera, Anabel Campos Garcia, Carmen Peiró Vilaplana

SWEDEN

Göteborg (Sahlgrenska University Hospital): Peter Berglund, Radu Constantinescu, Gunnel Fredlund, Ulrika Høsterey-Ugander, Petra Linnsand, Liselotte Neleborn-Lingefjärd, Jan Wahlström, Magnus Wentzel

Umeå (Umeå University Hospital): Ghada Loutfi, Carina Olofsson, Eva-Lena Stattin, Laila Westman, Birgitta Wikström

SWITZERLAND

Bern: Jean-Marc Burgunder, Yanik Stebler **(Swiss HD Zentrum)**, Alain Kaelin, Irene Romero, Michael Schüpbach, Sabine Weber Zaugg **(Zentrum für Bewegungsstörungen, Neurologische Klinik und Poliklinik, Universität Bern)**

Zürich (Department of Neurology, University Hospital Zürich): Maria Hauer, Roman Gonzenbach, Hans H. Jung, Violeta Mihaylova, Jens Petersen

UNITED KINGDOM

Aberdeen (NHS Grampian Clinical Genetics Centre & University of Aberdeen): Roisin Jack, Kirsty Matheson, Zosia Miedzybrodzka, Daniela Rae, Sheila A Simpson, Fiona Summers, Alexandra Ure, Vivien Vaughan

Birmingham (The Barberry Centre, Dept of Psychiatry): Shahbana Akhtar, Jenny Crooks, Adrienne Curtis, Jenny de Souza (Keylock), John Piedad, Hugh Rickards, Jan Wright

Bristol (North Bristol NHS Trust, Southmead hospital): Elizabeth Coulthard, Louise Gethin, Beverley Hayward, Kasia Sieradzan, Abigail Wright

Cambridge (Cambridge Centre for Brain Repair, Forvie Site): Matthew Armstrong, Roger A. Barker, Deidre O'Keefe, Anna Di Pietro, Kate Fisher, Anna Goodman, Susan Hill, Ann Kershaw, Sarah Mason, Nicole Paterson, Lucy Raymond, Rachel Swain, Natalie Valle Guzman

Cardiff (Schools of Medicine and Biosciences, Cardiff University): Monica Busse, Cynthia Butcher, Jenny Callaghan, Stephen Dunnett, Catherine Clenaghan, Ruth Fullam, Olivia Handley, Sarah Hunt, Lesley Jones, Una Jones, Hanan Khalil, Sara Minster, Michael Owen, Kathleen Price, Anne Rosser, Jenny Townhill

Edinburgh (Molecular Medicine Centre, Western General Hospital, Department of Clinical Genetics): Maureen Edwards, Carrie Ho (Scottish Huntington´s Association), Teresa Hughes (Scottish Huntington´s Association), Marie McGill, Pauline Pearson, Mary Porteous, Paul Smith (Scottish Huntington´s Association)

Fife (Scottish Huntington's Association Whyteman's Brae Hospital): Peter Brockie, Jillian Foster, Nicola Johns, Sue McKenzie, Jean Rothery, Gareth Thomas, Shona Yates

Gloucester (Department of Neurology Gloucestershire Royal Hospital): Liz Burrows, Carol Chu, Amy Fletcher, Deena Gallantrae, Stephanie Hamer, Alison Harding, Stefan Klöppel, Alison Kraus, Fiona Laver, Monica Lewis, Mandy Longthorpe, Ivana Markova, Ashok Raman, Nicola Robertson, Mark Silva, Aileen Thomson, Sue Wild, Pam Yardumian

Hull (Castle Hill Hospital): Carol Chu, Carole Evans, Deena Gallentrae, Stephanie Hamer, Alison Kraus, Ivana Markova, Ashok Raman

Leeds (Chapel Allerton Hospital, Department of Clinical Genetics): Leeds (Chapel Allerton Hospital, Clinical Genetics): Carol Chu, Stephanie Hamer, Emma Hobson, Stuart Jamieson, Alison Kraus, Ivana Markova, Ashok Raman, Hannah Musgrave, Liz Rowett, Jean Toscano, Sue Wild, Pam Yardumian

Leicester (Leicestershire Partnership Trust, Mill Lodge): Colin Bourne, Jackie Clapton, Carole Clayton, Heather Dipple, Dawn Freire-Patino, Janet Grant, Diana Gross, Caroline Hallam, Julia Middleton, Ann Murch, Catherine Thompson

Liverpool (Walton Centre for Neurology and Neurosurgery): Sundus Alusi, Rhys Davies, Kevin Foy, Emily Gerrans, Louise Pate

London (Guy's Hospital): Thomasin Andrews, Andrew Dougherty, Charlotte Golding, Fred Kavalier, Hana Laing, Alison Lashwood, Dene Robertson, Deborah Ruddy, Alastair Santhouse, Anna Whaite

London (The National Hospital for Neurology and Neurosurgery): Thomasin Andrews, Stefania Bruno, Karen Doherty, Charlotte Golding, Salman Haider, Davina Hensman, Nayana Lahiri, Monica Lewis, Marianne Novak, Aakta Patel, Nicola Robertson, Elisabeth Rosser, Sarah Tabrizi, Rachel Taylor, Thomas Warner, Edward Wild

Manchester (Genetic Medicine, University of Manchester, Manchester Academic Health Sciences Centre and Central Manchester University Hospitals NHS Foundation Trust): Natalie Arran, Judith

Bek, Jenny Callaghan, David Craufurd, Ruth Fullam, Marianne Hare, Liz Howard, Susan Huson, Liz Johnson, Mary Jones, Helen Murphy, Emma Oughton, Lucy Partington-Jones, Dawn Rogers, Andrea Sollom, Julie Snowden, Cheryl Stopford, Jennifer Thompson, Iris Trender-Gerhard, Nichola Verstraelen (formerly Ritchie), Leann Westmoreland

Oxford (Oxford University Hospitals NHS Trust, Dept. of Neurosciences, University of Oxford): Richard Armstrong, Kathryn Dixon, Andrea H Nemeth, Gill Siuda, Ruth Valentine

Plymouth (Plymouth Huntington Disease Service, Mount Gould Hospital): David Harrison, Max Hughes, Andrew Parkinson, Beverley Soltysiak

Sheffield (The Royal Hallamshire Hospital– Sheffield Children's Hospital): Oliver Bandmann, Alyson Bradbury, Paul Gill, Helen Fairtlough, Kay Fillingham, Isabella Foustanos, Mbombe Kazoka, Kirsty O'Donovan, Nadia Peppa, Cat Taylor, Katherine Tidswell, Oliver Quarrell

EHDN's associate site in Singapore: National Neuroscience Institute Singapore: Jean-Marc Burgunder, Puay Ngoh Lau, Emmanul Pica, Louis Tan

EHDN Behavioural Modifiers Working Group members

Aad Tibben, Akshay Nair, Andrea Higgins, Andrea Horta, Arvid Heiberg. Asa Peterson, Asunción Martínez Descals, Bernhard Landwehrmeyer, Carolyn Drazinic, Charlotte Goodwin, Cheryl Stopford, Clare Eddy, Constantine Economides, Daniel P. Van Kammen, David Craufurd, Dawn Freire-Patino, Ashraful Bari, Duncan McLauchlan, Emilia Sitek, Erik van Duijn, Fiona Eccles, George El-Nimr, Hanneke Nijsten, Hugh Rickards, Ida Unmack Larsen, Inga Bjørnevoll, James Osborne, Jan Van den Stock, Jan Wright, Jane Simpson, Jennifer Hoblyn, Jenny Callaghan, Jenny De Souza, Jesús Miguel Ruiz Idiago, Jesus Perez Perez, John Maltby, Josef Priller, Karen Anderson, LaVonne Goodman, Lesley Jones, Maria Dale, Marleen van Walsem, Martin Kucharík, Mary Edmondson, Matthias Dose, Mayke Oosterloo, Nicola Robertson, Noora Ovaska, Olga Klempirova, Oliver Tuscher, Pavel Ressner, Philipp Honrath, Rainer Hoffmann, Rebecca Fuller, Saül Martinez Horta, Tereza Uhrova, Tua Vinther-Jensen, Katie Osborne-Crowley, Natascia De Lucia, Marianna Delussi
